# Successful revascularization versus medical therapy in diabetic patients with stable right coronary artery chronic total occlusion: a retrospective cohort study

**DOI:** 10.1186/s12933-019-0911-4

**Published:** 2019-08-21

**Authors:** Yunfeng Yan, Mingduo Zhang, Fei Yuan, Hong Liu, Di Wu, Yudong Fan, Xinjing Guo, Feng Xu, Min Zhang, Quanming Zhao, Shuzheng Lyu

**Affiliations:** 1 Department of Cardiology, Beijing Anzhen Hospital, Capital Medical University, Beijing Institute of Heart, Lung and Blood Vessel Diseases, 2 Anzhen Road, Chaoyang District, Beijing, 100029 China; 2 Department of Cardiology, Emergency General Hospital, 29 Xibahe Nanli, Chaoyang District, Beijing, China

**Keywords:** Chronic total occlusion, Diabetes, Revascularization, Medical therapy

## Abstract

**Background:**

The territory of the right coronary artery (RCA) is smaller than that of the left anterior descending artery. Previous studies have reported conflicting results when considering whether stable RCA-chronic total occlusion (CTO) should be reopened. The coexistence of diabetic and coronary artery diseases represents a severe situation. Therefore, we aimed to determine if stable RCA-CTO in diabetic patients was necessary to be reopened. To our knowledge, no studies have focused on this topic to date.

**Methods:**

We enrolled diabetic patients with RCA-CTO who had clinical presentations of symptomatic stable angina or silent ischemia. RCA-CTO was treated with either successful revascularization (the CTO-SR group) or medical therapy (the CTO-MT group). The primary endpoint was all-cause death. Both Cox regression and propensity score matching analyses were used. Sensitivity analysis was performed based on subgroup populations and relevant baseline variables.

**Results:**

A total of 943 patients were included: 443 (46.98%) patients in the CTO-MT group and 500 (53.02%) patients in the CTO-SR group. After a mid-term follow-up (CTO-SR: 48 months; CTO-MT: 42 months), we found that CTO-SR was superior to CTO-MT in terms of all-cause death (adjusted hazard ratio [HR] [model 1]: 0.429, 95% conference interval [CI] 0.269–0.682; adjusted HR [model 2]: 0.445, 95% CI 0.278–0.714). The superiority of CTO-SR was consistent for cardiac death, possible/definite cardiac death, repeat revascularization, target vessel revascularization (TVR) and repeat nonfatal myocardial infarction. Subgroup analysis confirmed the mortality benefit of CTO-SR by percutaneous coronary intervention (the successful CTO-PCI subgroup, 309 patients in total). While CTO-SR by coronary artery bypass grafting (the CTO-CABG subgroup, 191 patients in total) offered patients more benefit from repeat revascularization and TVR than that offered by successful CTO-PCI.

**Conclusions:**

For stable RCA-CTO patients with diabetes, successful revascularization offered patients more clinical benefits than medical therapy. CTO-CABG might be a more recommended way to accomplish revascularization.

*Trial registration* This study was not registered in an open access database

**Electronic supplementary material:**

The online version of this article (10.1186/s12933-019-0911-4) contains supplementary material, which is available to authorized users.

## Background

The incidence of chronic total occlusion (CTO) is reported to reach 18.2–52% [1–4] in patients undergoing coronary angiography. Right coronary artery (RCA) CTO has been observed in approximately 38–50% [1, 5–7] of the entire CTO population. Although CTO revascularization was observed to have an acceptable success rate and to gain more clinical benefits from successful procedures [8, 9], the RCA-CTO patients who are treated with revascularization, either by percutaneous coronary intervention (PCI) or coronary artery bypass grafting (CABG), are fewer than those patients with left anterior descending branch (LAD) CTO [1, 3, 4, 10]. We speculated that the reason for this discrepancy might partially because the territory of the RCA is smaller than that of the LAD and partially because the conflicting debates on the question of whether the successful reperfusion of RCA-CTO offers patients clinical benefits. Safley and colleagues [5] enrolled the entire CTO population and reported that successful PCI for RCA-CTO did not offer patient survival benefits, which was also confirmed by other experts [11]. However, this claim was doubted by Mitomo and colleagues [6], who observed that successful RCA-CTO PCI was related to a lower cardiac mortality than that related to failed CTO-PCI. Interestingly, we observed that the percentages of diabetic patients in the three studies were different: 21%, 21.6% and 45.2%. These results implied that diabetic RCA-CTO patients might be different. Moreover, Migliorini and colleagues [10] demonstrated that the existence of RCA CTO is a significant risk predictor of death in patients with unprotected left main coronary artery disease who have undergone PCI, which indicated that RCA-CTO should be reopened in patients with severe coronary atherosclerosis in other vessels to prevent adverse events in cases of occlusion in a non-CTO epicardial artery.

Diabetes mellitus is observed in 34–40% CTO patients [12, 13]. The coexistence of diabetes is often associated with a severe coronary situation, which is characterized by endothelial cell dysfunction, microcirculation disorders, high-grade coronary atherosclerosis, a rapid progression of atherosclerosis and poor clinical outcomes [14–18]. Previous studies demonstrated clinical benefits after successful CTO revascularization [12, 19, 20]. However, to our knowledge, no study has focused on the diabetic RCA-CTO population.

Thus, in the present study, we focused on diabetic RCA-CTO patients who had clinical manifestations of stable angina or silent ischemia. We attempted to determine whether successful revascularization offers a clinical benefit when compared with conservative medical therapy.

## Methods

### Study design and population

The present study was a retrospective cohort study that was performed at Beijing Anzhen Hospital (Beijing, China). From January 2007 to December 2017, a population of 2502 stable patients with one main stem CTO were selected. In this study, we enrolled patients who had only RCA-CTO. The inclusion criteria were as follows: (1) diabetic patients with only one main stem CTO that was diagnosed by angiography and (2) patients with RCA-CTO (American Heart Association segment maps 1, 2 and 3) who had stable clinical presentations of symptomatic stable angina or silent ischemia. We excluded those patients with (1) a prior CABG history; (2) left main coronary artery disease (stenoses ≥ 50%); (3) a history of myocardial infarctions (MIs) due to a non-CTO artery within 30 days; and (4) patients with cancer or other diseases that may have confounded the endpoints.

Patients were assigned to the medical therapy (CTO-MT) group or to the successful revascularization (CTO-SR) group according to their final treatment strategy of the CTO vessel. Of note, the CTO-SR group enrolled patients who were treated with CTO-CABG or with successful CTO-PCI. The CTO-MT group enrolled patients who were managed by failed CTO-CABG, failed CTO-PCI or initial medical therapy of the CTO vessel. The definition of successful CTO-PCI was obtaining a residual stenosis of < 20% and a thrombolysis in myocardial infarction (TIMI) flow ≥ 2 [21] after implantation of a drug eluting stent to the CTO vessel. Failed CTO-CABG was defined as performing grafts to other arteries but not the RCA territory.

An optimal medical treatment was recommended to both groups of patients who were managed by SR and MT. To reiterate, an antiplatelet therapy involving the use of aspirin and/or clopidogrel (ticagrelor), a lipid lowering therapy, an antianginal therapy and other secondary prevention therapies were all recommended to be prescribed for the patients as needed.

The strategies of MT or revascularization (CABG or PCI) were both alternative methods for treating a CTO vessel, and the final strategy decision was made based on the preferences of both the physicians and the patients. During the PCI procedure, the uses of contemporary techniques, such as microcatheters, bilateral injections and retrograde approaches, among others, were left to the discretion of the operator.

### Definitions of variables and clinical endpoints

The definition of RCA-CTO was a TIMI flow grade of 0 within the RCA vessel with a duration of at least 3 months [22]. The occlusion interval was calculated from the last episode of myocardial infarction, from the first episode of stable angina or from a previous angiogram. For those patients who exhibited no clinical symptoms, we artificially identified them as meeting our inclusion criteria. Diabetes mellitus was defined as a previous diagnosis of diabetes before hospitalization or a new diagnosis with either fasting blood glucose levels ≥ 7.0 mmol/L or a glucose level at 2 h after a meal of ≥ 11.1 mmol/L on more than two occasions [23]. A prior MI was confirmed according to the electrocardiogram, the ultrasonic cardiogram or the records from a hospital information system. An angiographic stenosis > 50% was considered to indicated a diseased vessel. Patients with a CTO vessel that represented the only diseased vessel were diagnosed with single-vessel disease. Systolic heart failure consisted of only heart failure with a reduced ejection fraction (HFrEF) and heart failure with a mid-range ejection fraction, which was defined as a left ventricular ejection fraction (LVEF) < 50% with either the presence of dyspnea or equivalent symptoms, while diastolic heart failure exhibited a preserved ejection fraction (HFpEF) [24]. Both right dominance and codominance were considered to be right dominance.

We predefined the primary clinical endpoint as all-cause death, which was mortality due to any reason. Other clinical outcomes included cardiac death, repeat nonfatal MI, repeat revascularization and target vessel revascularization (TVR). Cardiac death was defined as the following by the Academic Research Consortium (ARC) [25]: any death that is related to a cardiac reason, an unwitnessed mortality or an unknown cause of mortality. Noncardiac death was defined as death from a certain noncardiac cause. Probable/definite cardiac death was defined as death of certain (myocardial infarction, heart failure, etc.) or probable (sudden death) causes. The definition of a repeat MI was determined by following the third universal definition of MI [26]: a complicated consideration of ischemic symptoms, electrocardiograms and myocardial damage biomarkers. In this study, we collected data from only repeat nonfatal MI patients. The definition of repeat revascularization was an unplanned revascularization to the target vessel (RCA) or to the nontarget vessel. TVR was defined as any surgical bypass or percutaneous intervention of the target vessel (RCA).

### Data collection and follow-up

The data collection was managed by experienced raters who were trained beforehand in order to ensure accordance. The following items were selected for analysis: age, gender, clinical history (hypertension, dyslipidaemia, prior MI and systolic heart failure, among others), inspection information, medical data and angiogram information. For the angiogram data, experienced, interventional physicians rescanned the cine angiograms by using standard definitions in order to reduce bias.

A minimum follow-up period of 12 months was predefined. A pre-designed chart including all of the follow-up items was applied. The follow-up procedure was performed by experienced investigators who were blind to the patients’ assigned groups. A phone call was the preferred follow-up method. For patients who had records of re-hospitalization in the Beijing Anzhen Hospital, noteworthy information was also obtained from the hospital information system. In the present study, we attempted to minimise the crossover between the groups. Thus, for patients who changed their treatment strategy during the follow-up, we ended the follow-up process at the moment of the treatment change. The follow-up information was evaluated by an adjudication board (Shuzheng Lyv, Hong Liu and Fei Yuan) who were blind to the patients’ assigned groups. The certifications of the clinical endpoints were also based on the decisions of the board.

### Statistical analysis

The continuous variables are presented as the means ± SDs (normal distribution) or medians with interquartile ranges (skewed distribution). Comparisons were performed using Student’s *t*-test or the Mann–Whitney U test, where appropriate. The categorical items are presented as numbers and percentages and were analyzed using the Chi-square test or Fisher’s exact text. Events per 1000 patient-years and survival curves using the Kaplan–Meier method were applied for all of the endpoints. A univariate Cox proportional hazard regression model was used to calculate the unadjusted hazard ratios (HRs). After the univariable analysis, a multivariate Cox regression model was performed. The potential adjusted factors were selected according to both the univariate Cox regression model (via the analysis of all of the variables listed in Table 1 and by using a threshold of P ≤ 0.2) and the relevant clinical implications. The adjusted HR (model 1) was calculated by utilizing the variables that exhibited statistical significance, as demonstrated by the univariate Cox regression: age, chronic kidney disease (CKD), chronic obstructive pulmonary disease (COPD)/asthma, prior MI, systolic heart failure (HF), LVEF, regional wall motion abnormality (RWMA), single-vessel disease, triple-vessel disease and syntax scores. Afterwards, we added further potential clinically relevant factors to calculate the adjusted HR (model 2): sex, peripheral vascular disease (PVD) and HbA1c. To further evaluate the difference between the CTO-SR and CTO-MT groups, we performed a propensity score-matched analysis. First, a propensity score utilizing a logistic regression model, which absorbed all of the variables listed in Table 1 (except for the retrograde approach), was calculated. The pairs were then matched at a 1:1 ratio by utilizing a nearest-neighbor matching method (caliper value = 0.02). We assessed the balance of the variables through absolute standardized differences (ASDs). ASDs < 10.0% revealed a relatively small imbalance. The baseline items of the propensity-matched population were then reanalyzed using the same methods as previously described. The clinical endpoints were also reanalyzed with the Kaplan–Meier method and the log-rank tests. The univariate Cox proportional hazard regression model was applied to calculate the HRs. To evaluate whether right coronary dominance affects clinical endpoints, an additional analysis of patients with only right dominance was also performed between the CTO-SR and CTO-MT groups.

**Table 1:** Baseline characteristics (total population n = 943)

	CTO-MT (n = 443)	CTO-SR (n = 500)	P value
Clinical characteristics			
Age (years)	60.65 ± 10.58	59.97 ± 8.78	0.285
Male	322 (72.7)	392 (78.4)	*0.041*
Hypertension	308 (69.5)	337 (67.4)	0.483
Dyslipidemia	152 (34.3)	139 (27.8)	*0.031*
PVD	18 (4.1)	15 (3.0)	0.375
Prior MI	234 (52.8)	285 (57.0)	0.198
Prior PCI	90 (20.3)	72 (14.4)	*0.016*
Prior stroke	35 (7.9)	22 (4.4)	*0.024*
Heart failure	125 (28.2)	149 (29.8)	0.593
Systolic heart failure	52 (11.7)	63 (12.6)	0.686
Diastolic heart failure	73 (16.5)	86 (17.2)	0.768
CKD	15 (3.4)	10 (2.0)	0.186
COPD/asthma	4 (0.9)	4 (0.8)	0.864
Hyperuricemia	97 (21.9)	86 (17.2)	*0.069*
Smoking	216 (48.8)	266 (53.2)	0.173
Drinking	74 (16.7)	91 (18.2)	0.546
BMI (kg/m^2^)	26.46 ± 3.31	26.67 ± 3.02	0.304
LVEF (%)	61.00 (55.00–66.50)	60.00 (55.00–66.00)	0.153
RWMA	144 (32.5)	160 (32.0)	0.868
Fasting blood glucose(mmol/L)	7.20 (6.25–9.31)	7.41 (6.13–9.26)	0.727
HbA1c (%)^b^	7.3 (6.7–8.0)	7.3 (6.9–7.9)	0.753
Medical treatment			
Aspirin	428 (96.8)	429 (98.4)	0.114
P2Y_12_ inhibitor	396 (89.6)	410 (82.3)	*0.001*
Statin	422 (95.5)	462 (92.8)	0.080
Nitrites	262 (59.3)	152 (30.5)	*0.000*
Beta-blocker	341 (77.1)	405 (81.3)	0.114
CCB	123 (27.8)	123 (24.7)	0.276
ACEI/ARB	246 (55.7)	272 (54.6)	0.750
Insulin	160 (36.1)	202 (40.4)	0.177
Sulfonylureas	64 (14.4)	66 (13.2)	0.579
Glinide	20 (4.5)	18 (3.6)	0.476
Biguanides	152 (34.3)	195 (39.0)	0.136
Thiazolidinediones	48 (10.8)	54 (10.8)	0.986
Alpha-glucosidase inhibitor	159 (35.9)	151 (30.2)	0.063
Angiographic characteristics			
Dominance artery (right)	396 (89.4)	488 (97.6)	*0.000*
Number of diseased vessels			
1	115 (26.0)	94 (18.8)	*0.008*
2	151 (34.1)	201 (40.2)	0.053
3	177 (40.0)	205 (41.0)	0.744
Syntax score^a^	20.00 (13.00–27.00)	20.00 (17.00–23.00)	0.400
Rentrop grade ≥ 2^a^	309 (82.4)	368 (88.0)	*0.025*
Abrupt stump^a^	188 (50.1)	205 (49.0)	0.759
Calcification^a^	73 (19.5)	80 (19.1)	0.907
Bending ≥ 45°^a^	327 (87.2)	361 (86.4)	0.729
CTO length ≥ 20 mm^a^	278 (74.1)	308 (73.7)	0.886
Procedural characteristics			
Retrograde approach^c^	3 (1.6)	41 (13.3)	*0.000*
Perforation^c^	4 (0.9)	1 (0.2)	0.193
Pericardial effusion^c^	0 (0)	0 (0)	–
Emergency surgery^c^	0 (0)	0 (0)	–
Contrast retention/dissection^c^	2 (0.5)	5 (1.0)	0.457
Thread off^c^	1 (0.2)	0 (0)	0.470
Sudden cardiac arrest^c^	0 (0)	1 (0.2)	1.000
Death during hospitalization^c^	1 (0.2)	3 (0.6)	0.627

Values are n (%), mean ± SD or median with interquartile range

*PCI* percutaneous transluminal coronary intervention, *MT* medical therapy, *CABG* coronary artery bypass grafting, *PVD* peripheral vascular disease, *MI* myocardial infarction, *CKD* chronic kidney disease, *COPD* chronic obstructive pulmonary disease, *LVEF* left ventricular ejection fraction, *BMI* body mass index, *CCB* calcium-channel blocker, *ACEI/ARB* angiotensin converting enzyme inhibitor/angiotensin-receptor blocker; *CTO* chronic total occlusion, *HF* heart failure, *RWMA* reginal wall motion abnormality

^a^Cine angiograms records got from 794 (84.10%) individuals

^b^HbA1c got from 896 (95.02%) individuals

^c^Only patients who were treated with PCI

Subgroup analysis was performed between subgroup populations: successful CTO-PCI versus initial CTO-MT, successful CTO-PCI versus failed CTO-PCI, successful CTO-PCI versus CTO-CABG, CTO-CABG versus initial CTO-MT and CTO-CABG versus failed CTO-PCI.

Another subgroup analysis, including sex (male/female), age (< 60 years old/≥ 60 years old), prior MI (yes/no), RWMA (yes/no), HbA1c (< 7.0%/≥ 7.0%), single-vessel disease (yes/no), systolic heart failure (yes/no), Rentrop grade ≥ 2 (yes/no) and syntax score (< 22/≥ 22), was also conducted using a multivariate Cox regression model. The covariates that were applied in this Cox regression model were the same as the items applied in model 2. Of note, we only performed this subgroup analysis for all-cause death and cardiac death.

Statistical analyses were performed using SPSS 24.0 (SPSS Inc., Chicago, Illinois, USA) and Stata 14.0 (Stata, College Station, TX, USA). A two-tailed P-value ≤ 0.05 was considered statistically significant.

## Results

### Baseline characteristics (total population)

**Fig. 1:**
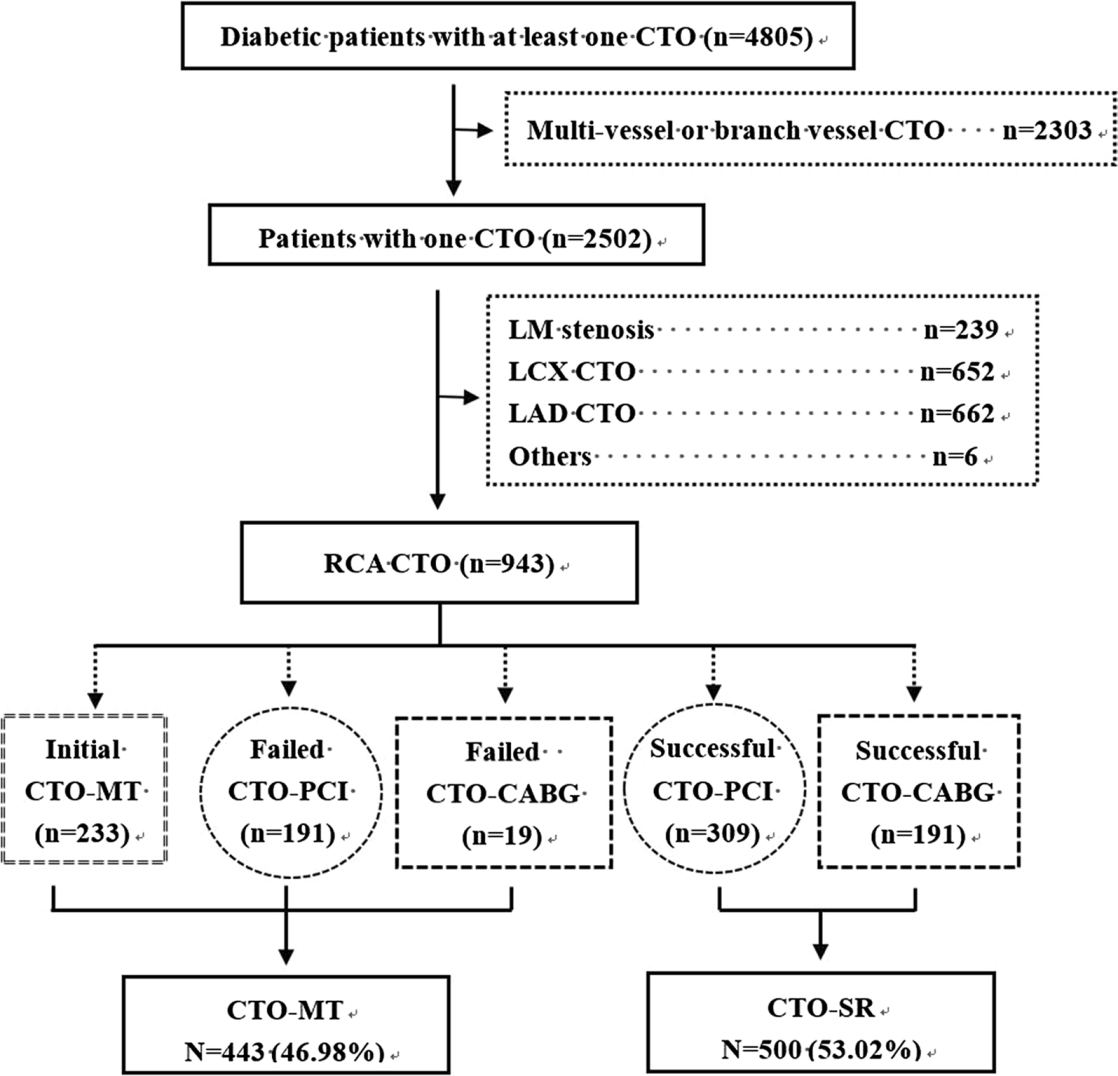
Flow chart of the present study

From January 2007 to December 2017, a total of 943 stable RCA-CTO patients with diabetes were consecutively enrolled in the present study (Fig. 1). Of these patients, 443 (46.98%) were managed by MT (the CTO-MT group), and 500 (53.02%) were managed by successful revascularization (the CTO-SR group). The CTO-MT group enrolled patients with initial CTO-MT (n = 233), failed CTO-PCI (n = 191) and failed CTO-CABG (n = 19). The CTO-SR group enrolled patients with successful CTO-PCI (n = 309) and CTO-CABG (n = 191).

The baseline clinical and angiographic characteristics are listed in Table 1. To reiterate, compared to those in the CTO-SR group, patients who were treated with MT had a higher prevalence of dyslipidemia, hyperuricemia, prior PCI, prior stroke, statin intake, nitrite intake and single-vessel disease, whereas these patients were less likely to be male and had a lower prevalence of right dominance coronary circulation and good collateral circulation (Rentrop grade ≥ 2). Patients who were treated with SR were more likely to undergo the retrograde approach. For the other variables, there were no significant differences between the groups.

### Clinical outcomes

In-hospital mortality occurred in four patients, and these patients were all treated with CABG: three patients in the CTO-SR group and one patient in the CTO-MT group. When analyzing the mid-term clinical endpoints, we excluded these four patients. Procedural complications occurred in seven patients who were treated with failed CTO-PCI (the CTO-MT group): four patients exhibited contrast extravasation (no pericardial tamponade), two patients with coronary artery dissection, and the guidewire broke and remained in the distal iliac artery in one patient. Additionally, complications occurred in seven patients who were managed by successful CTO-PCI: one patient exhibited contrast extravasation (no pericardial tamponade), five patients had contrast retention and one patient experienced sudden cardiac arrest during the procedure.

Table 2 shows the clinical endpoints of this study. After a mid-term follow-up (CTO-MT: median: 42.00 months, interquartile range [IQR]: 24.00–78.25 months; CTO-SR: 48 [29.00–90.50] months), the data on all-cause death was successfully obtained from 874 (93.08%) patients: 28.15 all cause death per 1000 patient-years in the CTO-MT group versus 12.17 all cause death per 1000 patient-years in the CTO-SR group (P < 0.001). The univariate analysis (unadjusted HR: 0.423, 95% conference interval [CI] 0.270–0.663) demonstrated that CTO-SR was superior to CTO-MT for all-cause death. This superiority was also confirmed by the multivariate analysis (adjusted HR [model 1]: 0.429, 95% CI 0.269–0.682; adjusted HR [model 2]: 0.445, 95% CI 0.278–0.714).

**Table 2:** Clinical outcomes in all patients

	CTO-MT	CTO-SR	P value
All cause death			
Event per 1000 patient-years	28.05	12.17	*0.000*
Unadjusted HR (95% CI)	1	*0.423* (*0.270–0.663*)	*0.000*
Adjusted HR (95% CI) Model 1	1	*0.429* (*0.269–0.682*)	*0.000*
Adjusted HR (95% CI) Model 2	1	*0.445* (*0.278–0.714*)	*0.001*
Noncardiac death			
Event per 1000 patient-years	5.82	5.28	0.808
Unadjusted HR (95% CI)	1	0.829 (0.370–1.860)	0.649
Adjusted HR (95% CI) Model 1	1	1.351 (0.538–3.392)	0.522
Adjusted HR (95% CI) Model 2	1	1.778 (0.684–4.618)	0.237
Cardiac death			
Event per 1000 patient-years	22.23	6.90	*0.000*
Unadjusted HR (95% CI)	1	*0.311* (*0.177–0.547*)	*0.000*
Adjusted HR (95% CI) Model 1	1	*0.308* (*0.172–0.550*)	*0.000*
Adjusted HR (95% CI) Model 2	1	*0.307* (*0.169–0.557*)	*0.000*
Probable/definite cardiac death			
Event per 1000 patient-years	12.17	3.65	*0.000*
Unadjusted HR (95% CI)	1	*0.297* (*0.137–0.644*)	*0.002*
Adjusted HR (95% CI) Model 1	1	*0.309* (*0.138–0.693*)	*0.004*
Adjusted HR (95% CI) Model 2	1	*0.341* (*0.151–0.771*)	*0.010*
Repeat nonfatal MI			
Event per 1000 patient-years	13.58	7.00	*0.031*
Unadjusted HR (95% CI)	1	*0.493* (*0.266–0.915*)	*0.025*
Adjusted HR (95% CI) Model 1	1	*0.466* (*0.245–0.888*)	*0.020*
Adjusted HR (95% CI) Model 2	1	*0.513* (*0.264–0.998*)	*0.049*
Repeat revascularization			
Event per 1000 patient-years	56.95	37.86	*0.005*
Unadjusted HR (95% CI)	1	*0.683* (*0.509–0.915*)	*0.011*
Adjusted HR (95% CI) Model 1	1	*0.689* (*0.512–0.928*)	*0.014*
Adjusted HR (95% CI) Model 2	1	*0.702* (*0.516–0.955*)	*0.024*
TVR			
Event per 1000 patient-years	36.13	20.48	*0.002*
Unadjusted HR (95% CI)	1	*0.591* (*0.406–0.862*)	*0.006*
Adjusted HR (95% CI) Model 1	1	*0.587* (*0.401–0.858*)	*0.006*
Adjusted HR (95% CI) Model 2	1	*0.613* (*0.413–0.909*)	*0.015*

Adjusted covariates (model 1): age, CKD, COPD/asthma, prior MI, systolic HF, LVEF, reginal wall motion abnormality, single vessel disease, triple-vessel disease and syntax scores

Adjusted covariates (model 2): age, sex, PVD, CKD, COPD/asthma, prior MI, systolic HF, LVEF, reginal wall motion abnormality, single vessel disease, triple-vessel disease, syntax scores and HbA1c

*HR* hazard ratio, *CI* conference interval; other abbreviations as in Table 1

**Fig. 2:**
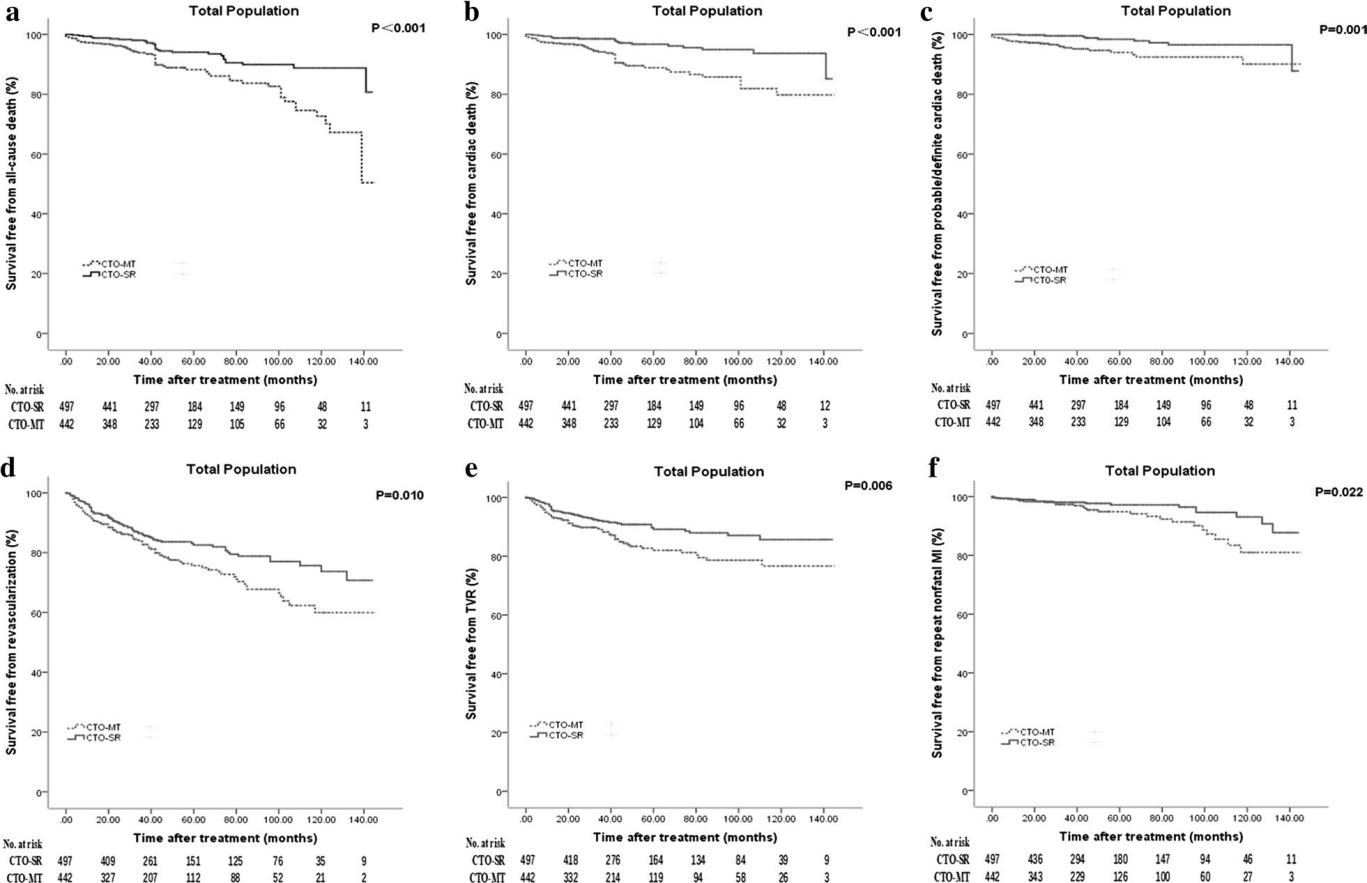
Kaplan–Meier curves for clinical endpoints in all patients. **a** Kaplan–Meier curves for all cause death in patients treated with successful revascularization versus medical therapy; **b** Kaplan–Meier curves for cardiac death; **c** Kaplan–Meier curves for probable/definite cardiac death; **d** Kaplan–Meier curves for repeat revascularization; **e** Kaplan–Meier curves for target vessel revascularization (TVR); **f** Kaplan–Meier curves for repeat nonfatal MI

When considering the outcome of cardiac death, CTO-SR also demonstrated its superiority in events per 1000 patient-years (22.23 versus 6.90, P < 0.001), the univariate (unadjusted HR: 0.311, 95% CI 0.177–0.547) and multivariate (adjusted HR [model 1]: 0.308, 95% CI 0.172–0.550; adjusted HR [model 2]: 0.307, 95% CI 0.169–0.557) models. So as to probable/definite cardiac death (unadjusted HR: 0.297, 95% CI 0.137–0.644; adjusted HR [model 1]: 0.309, 95% CI 0.138–0.693; adjusted HR [model 2]: 0.341, 95% CI 0.151–0.771). The superiority of patients who were managed by SR was consistent for the clinical endpoints of repeat nonfatal MI (unadjusted HR: 0.493, 95% CI 0.266–0.915; adjusted HR [model 1]: 0.466, 95% CI 0.245–0.888; adjusted HR [model 2]: 0.513, 95% CI 0.264–0.998), repeat revascularization (unadjusted HR: 0.683, 95% CI 0.509–0.915; adjusted HR [model 1]: 0.689, 95% CI 0.512–0.928; adjusted HR [model 2]: 0.702, 95% CI 0.516–0.955) and TVR (unadjusted HR: 0.591, 95% CI 0.406–0.862; adjusted HR [model 1]: 0.587, 95% CI 0.401–0.858; adjusted HR [model 2]: 0.613, 95% CI 0.413–0.909) (Table 2 and Fig. 2).

### Propensity score matched analysis

**Fig. 3:**
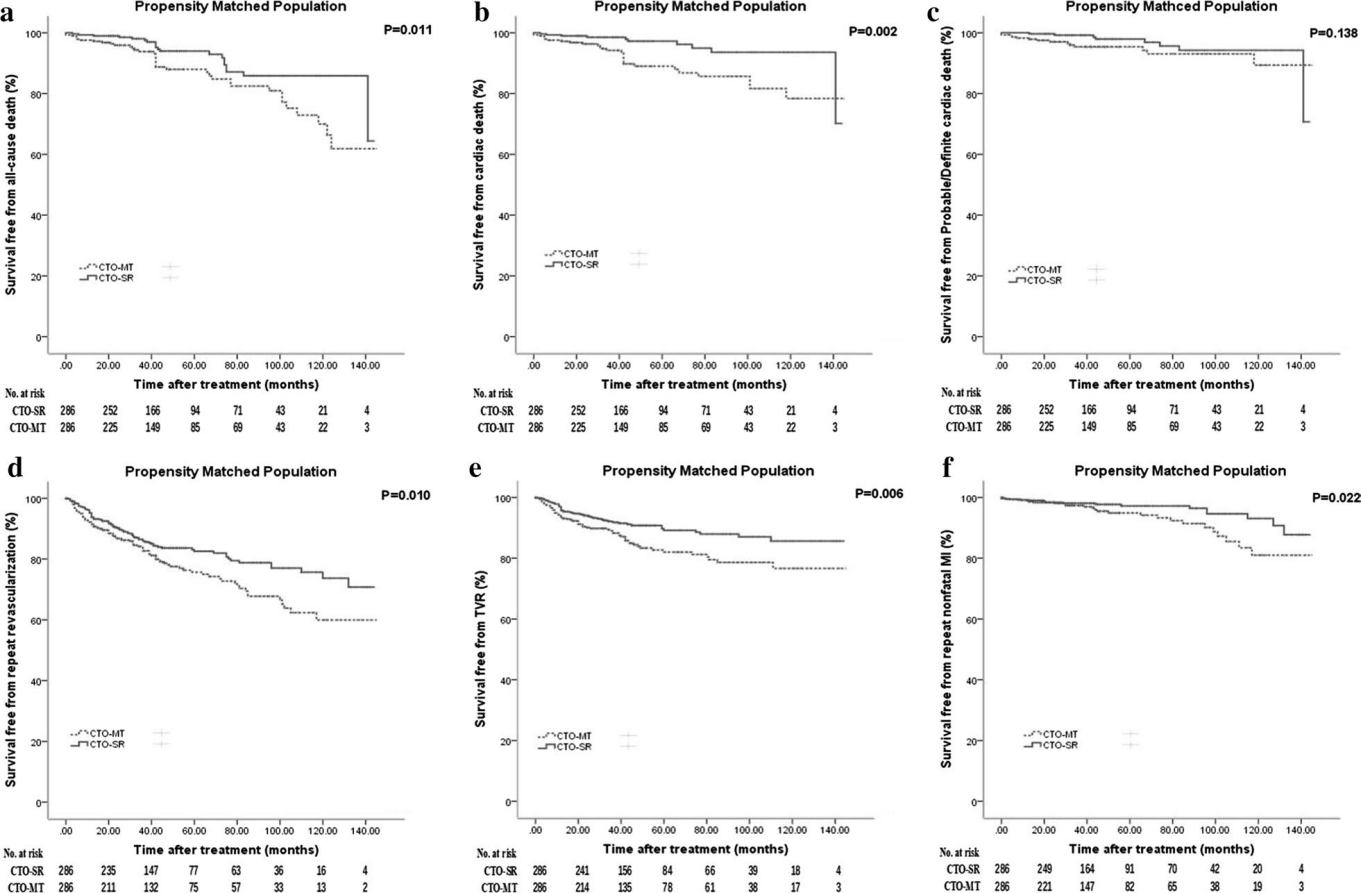
Kaplan–Meier curves for clinical endpoints in propensity-matched population. **a** Kaplan–Meier curves for all cause death in patients treated with successful revascularization versus medical therapy; **b** Kaplan–Meier curves for cardiac death; **c** Kaplan–Meier curves for probable/definite cardiac death; **d** Kaplan–Meier curves for repeat revascularization; **e** Kaplan–Meier curves for target vessel revascularization (TVR); **f** Kaplan–Meier curves for repeat nonfatal MI

After a 1:1 propensity matching, 286 patients were included in both groups. The ASDs after matching were all less than 10.0%, except for drinking (see Additional file 1: Figure S1). None of the baseline variables showed significant differences (see Additional file 1: Table S1), thus indicating a good matching balance. For the clinical endpoints, the superiority of CTO-SR was also observed for all-cause death (HR: 0.502, 95% CI 0.291–0.865, P = 0.013), cardiac death (HR: 0.331, 95% CI 0.161–0.681, P = 0.003), repeat revascularization (HR: 0.639, 95% CI 0.440–0.927, P = 0.018) and TVR (HR: 0.574, 95% CI 0.355–0.930, P = 0.024) when compared with CTO-MT (Table 3 and Fig. 3). Regarding repeat nonfatal MI, the trend was also consistent (HR: 0.571, 95% CI 0.277–1.177, P = 0.129).

**Table 3:** Clinical outcomes in propensity matched population

	CTO-MT	CTO-SR	P value
All cause death			
Event per 1000 patient-years	30.15	15.03	*0.010*
HR (95% CI)	1	*0.502* (*0.291–0.865*)	*0.013*
Non-cardiac death			
Event per 1000 patient-years	7.34	7.52	0.957
HR (95% CI)	1	1.038 (0.422–2.555)	0.936
Cardiac death			
Event per 1000 patient-years	22.82	7.52	*0.001*
HR (95% CI)	1	*0.331* (*0.161–0.681*)	*0.003*
Probable/definite cardiac death			
Event per 1000 patient-years	11.41	6.01	0.140
HR (95% CI)	1	0.524 (0.220–1.250)	0.145
Repeat nonfatal MI			
Event per 1000 patient-years	15.95	9.16	0.126
HR (95% CI)	1	0.571 (0.277–1.177)	0.129
Repeat revascularization			
Event per 1000 patient-years	61.79	39.08	*0.012*
HR (95% CI)	1	*0.639* (*0.440–0.927*)	*0.018*
TVR			
Event per 1000 patient-years	37.70	21.45	*0.018*
HR (95% CI)	1	*0.574* (*0.355–0.930*)	*0.024*

*HR* hazard ratio, *CI* conference interval; other abbreviations as in Table 1

### Subgroup analysis

**Fig. 4:**
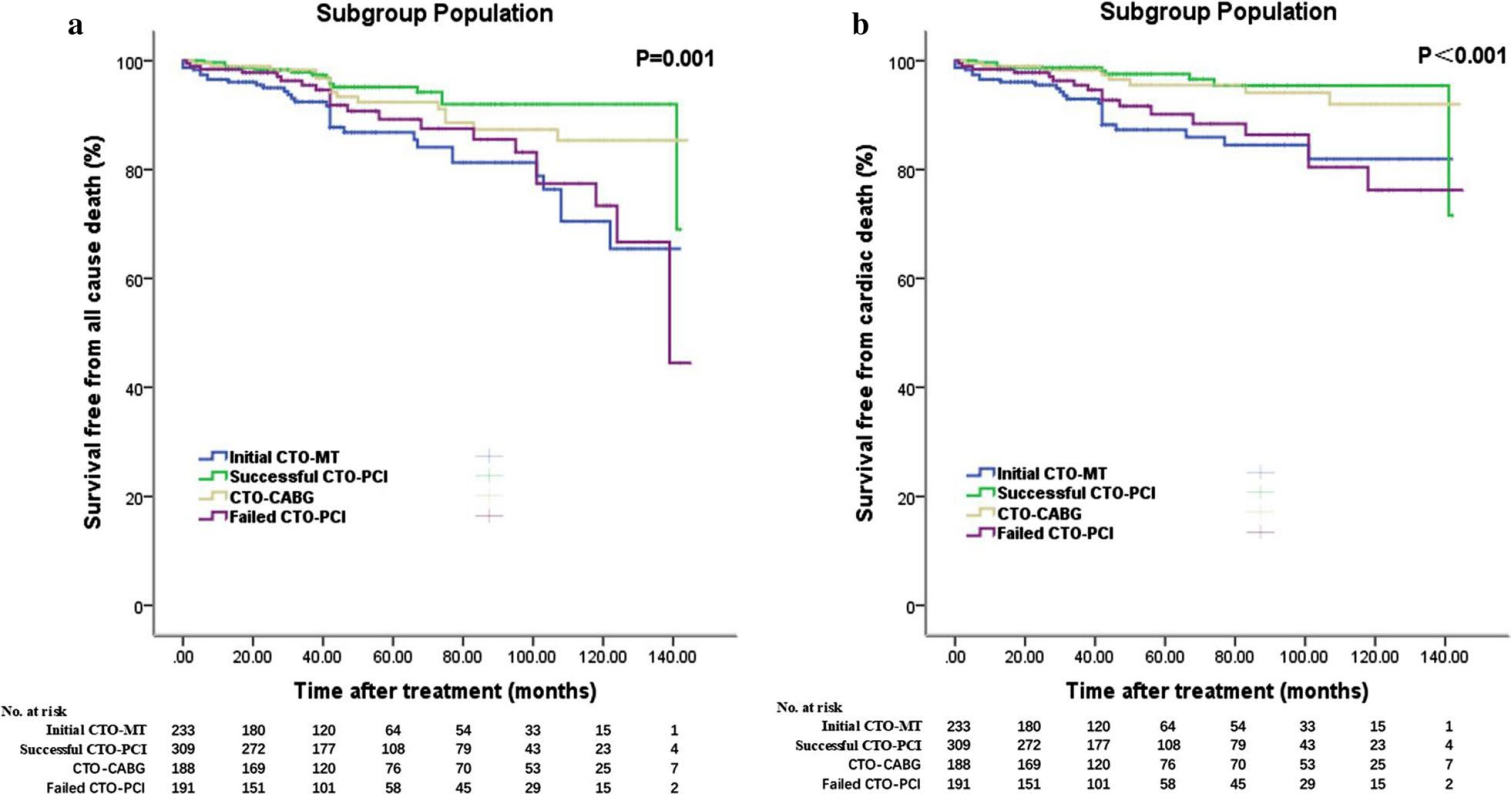
Kaplan–Meier curves for clinical endpoints in subgroup population. **a** Kaplan–Meier curves for all cause death in patients treated with initial CTO-MT versus successful CTO-PCI versus failed CTO-PCI versus CTO-CABG; **b** Kaplan–Meier curves for cardiac death

To minimize the influence of the dominance artery, an additional analysis that included only patients with RAC dominance was performed. We found that the superiority of CTO-SR was consistent in all clinical endpoints as in the whole population (see Additional file 1: Table S3, Fig. 4). The baseline characteristics are also provided in Additional file 1: Table S2.

Another subgroup analysis that compared successful CTO-PCI with failed CTO-PCI, initial CTO-MT and CTO-CABG, found that successful CTO-PCI provided patients with a benefit in terms of mortality when compared with initial CTO-MT (all-cause death: adjusted HR 0.498 [0.251–0.987]) and failed CTO-PCI (all-cause death: adjusted HR 0.315 [0.154–0.642]). This benefit can mainly be attributed to cardiac causes (cardiac death: 0.371 [0.154–0.892] and 0.195 [0.082–0.465]; probable/definite cardiac death: 0.463 [0.126–1.701] and 0.151 [0.041–0.563], respectively) (see Additional file 1: Tables S4–S7, Fig. 4). Regarding successful CTO-PCI versus CTO-CABG, CTO-CABG gained more benefit from repeat revascularization (adjusted HR 4.459 [2.044–9.726]) and TVR (21.676 [2.896–162.24]) (see Additional file 1: Tables S8, S9, Fig. 4).

We also compared CTO-CABG with both initial CTO-MT and failed CTO-PCI. Although statistical significance was not observed in all endpoints, CTO-CABG was superior to both subgroups in all-cause death, cardiac death, repeat revascularization and TVR. (see Additional file 1: Table S10–S13, Fig. 4).

**Fig. 5:**
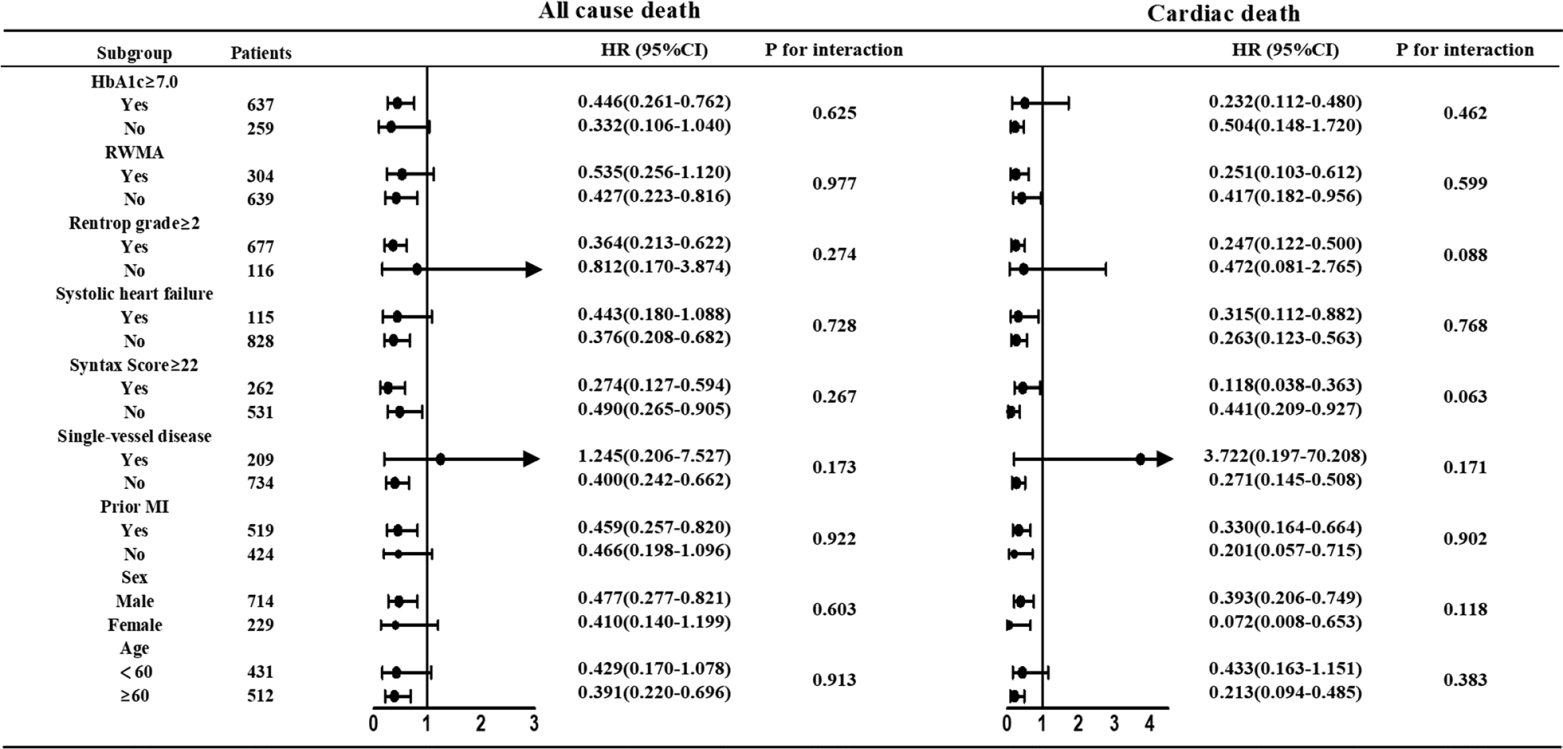
Subgroup analysis for all-cause death and cardiac death. All HRs were adjusted for age, sex, PVD, CKD, COPD/asthma, prior MI, systolic HF, LVEF, reginal wall motion abnormality, single vessel disease, triple-vessel disease, syntax scores and HbA1c (abbreviations as in Table 1)

Subgroup analysis based on clinically relevant variables showed that the superiority of CTO-SR in all-cause death was consistent in all subgroups, except for patients with single-vessel disease (HR: 1.245 [0.206–7.527]). The same results were also observed in cardiac death (Fig. 5). However, only 8 all-cause deaths and 4 cardiac deaths occurred in patients with single-vessel disease. Furthermore, no significant difference was observed in patients with/without single-vessel disease (P > 0.05).

### Predictors of survival

**Fig. 6:**
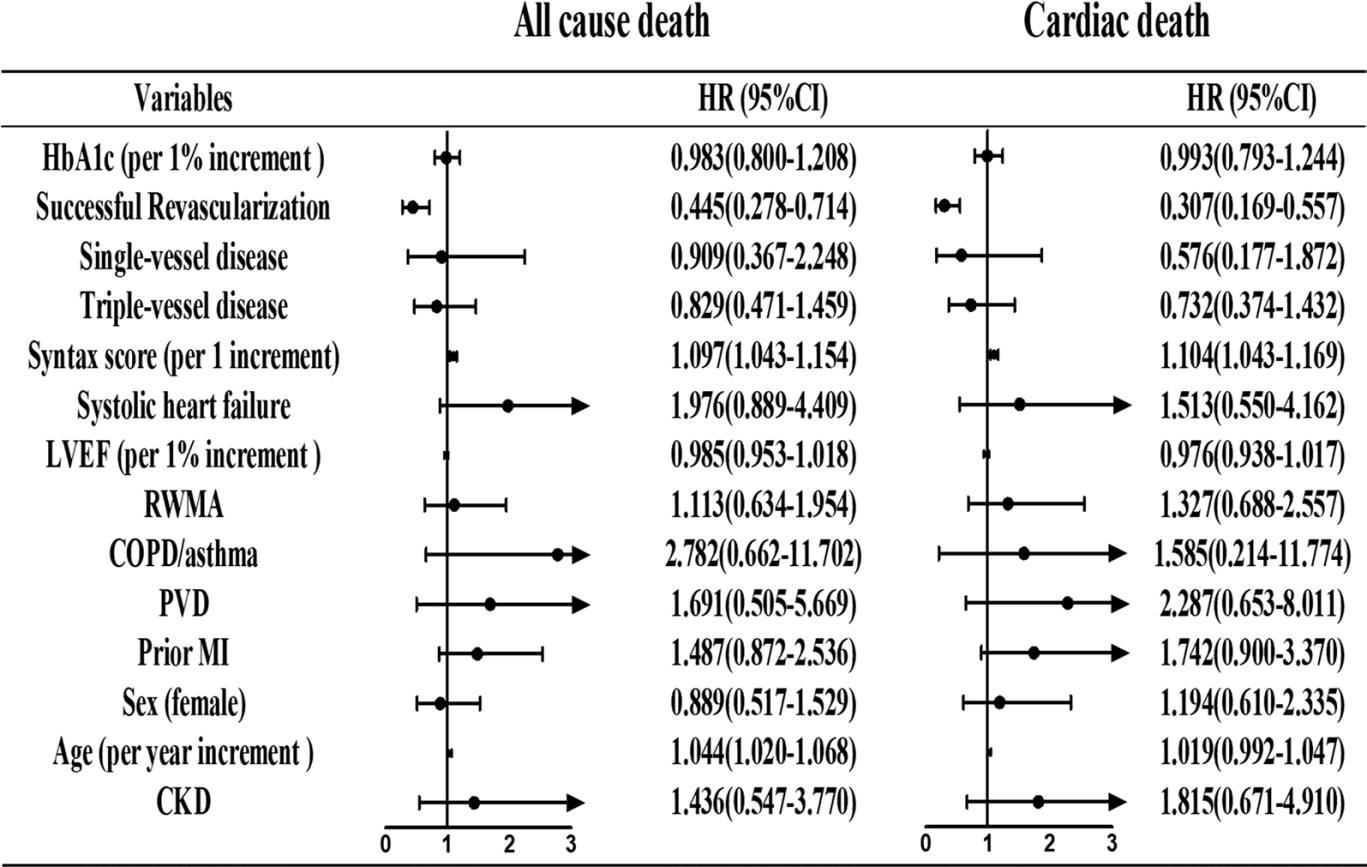
Predictors of all-cause death and cardiac death

By multivariate analysis (Fig. 6), CTO-SR was a protected predictor of all-cause death (HR: 0.445, 95% CI 0.278–0.714) and cardiac death (HR: 0.307, 95% CI 0.169–0.557). Conversely, increases in syntax score (per 1 score, HR: 1.097, 95% CI 1.043–1.154) and age (per 1 year, HR: 1.044, 95% CI 1.020–1.068) predicted a worse probability for all-cause death. Additionally, an increase in the syntax score (per 1 score, HR: 1.104, 95% CI 1.043–1.169) was also related to a higher incidence of cardiac death.

## Discussion

### Main discoveries

To our knowledge, this is the first study that focused on stable RCA-CTO patients with diabetes and that attempted to determine if SR for RCA-CTO offered patients better clinical benefit than that offered by conservative MT. After a mid-term follow-up, our main findings are as follows: (1) CTO-SR offered patients more clinical benefits, which was demonstrated by both the multivariable Cox regression analysis, a propensity score-matched analysis and the subgroup analyses. (2) The subgroup analyses revealed that mortality benefits come from both successful CTO-PCI and CTO-CABG. While, the benefit of repeat revascularization and TVR mainly comes from the CTO-CABG subgroup. Furthermore, CTO-CABG also offered patients more repeat revascularization and TVR benefits when compared with successful CTO-PCI. (3) Syntax score was a harmful predictor of cardiac death. However, neither collateral circulation nor syntax score influenced the survival benefits of CTO-SR. So as to glycemic control (HbA1c).

### Conflicting debates on RCA-CTO treatment strategy

It is well-known that the territory of the RCA is smaller than that of the LAD. Additionally, good collateral circulation has more often been observed in RCA-CTO [27]. Thus, some experts have argued that successful revascularization for RCA-CTO will not benefit patients. Previous studies [5, 11] reported that successful PCI for RCA-CTO did not offer patient survival benefits, which may provide support for this plausible notion. However, Kalandyk and colleagues [28] reported that RCA-CTO increased the incidence of major adverse cardiac cerebrovascular events in patients undergoing CABG, which might mainly due to the smaller number of grafts to the RCA, indirectly indicating that RCA-CTO should be treated with revascularization. Direct positive evidence was also provided by Mitomo and colleagues [6]. However, all of the above studies focused on the entire CTO population. Diabetic status was associated with fast atherosclerosis progression and poor clinical outcomes [18, 29–33]. Under such a special situation, whether revascularization should be performed is unknown. Our study demonstrated that CTO-SR offers this specific patient group both survival benefits and other clinical benefits (lower incidence of repeat revascularization, TVR and repeat nonfatal MI). Moreover, we found that successful CTO-PCI offers patients only a mortality benefit, while CTO-CABG offers patients more clinical benefits than successful CTO-PCI does. This will surely better direct clinical practice.

Regarding procedural complications, in the present study, we calculated complications mainly from the PCI procedure. The incidence was low: seven for successful CTO-PCI and seven for failed CTO-PCI. After follow-up, we observed only six instances of repeat-revascularization (all were TVR) in those patients (four patients with successful CTO-PCI and two patients with failed CTO-PCI). No death or other adverse events occurred. We thought the influence of procedural complications on mid-term clinical outcomes might be slight.

### Why should diabetic patients with RCA-CTO be any different?

Previous findings can provide some clues to explain this issue. Prior studies [10, 34] have focused on patients with both RCA-CTO and other severe situations (older age, unprotected left main artery) and reported a higher incidence of mortality or cardiac mortality. A smaller RCA (left coronary dominance) is also a predictor of all-cause death in CTO patients [35]. Thus, the importance of RCA will be more obvious in patients with other severe conditions.

When considering diabetes, the SWISSI study [36] demonstrated that diabetes was a predictor of progressive coronary atherosclerosis disease (OR: 19.01, P = 0.026). The DIABETES study [37] reported that 50% of repeat revascularization procedures were due to progression in another vessel that was not previously treated. This finding was also confirmed by other experts [38]. We speculated that RCA-CTO is different in patients with diabetes because it is an inherently severe situation and exhibits a relatively fast progression, even though the RCA area might be smaller than the LAD area. One study [39] examined diabetic patients with a non-infarct-related CTO who suffered a ST-elevation myocardial infarction and demonstrated that CTO increased the risk of mortality when compared with those without CTO, which partially confirmed our speculation. Moreover, diabetic patients suffered a higher risk of any cardiovascular event than the risk in patients without diabetes [17]. From that perspective, the aim of successful revascularization is not only to recover the blood supply of the RCA area but also to support potential collateral circulation to either the LAD or the left circumflex coronary artery (LCX) in the case of an acute coronary occlusion.

Another possible explanation is electronic stability. The VACTO Primary Study [40] demonstrated that CTO was an independent risk factor for predicting the incidence of ventricular arrhythmias. Perfusion therapy can decrease the incidence of fatal ventricular arrhythmias [41]. Thus, electronic stability might partially explain the improvement in survival after successful revascularization.

To summarize, the inherent severity of the coronary artery situation in diabetic patients may make RCA-CTO different in diabetic patients than in the traditional patient population. The highlight of our study was demonstrating the successful revascularization benefits of patients in terms of not only survival items but also other clinical items. Furthermore, we demonstrated that successful CTO-PCI mainly offers patient survival benefits.

### Will collateral circulation, coronary severity and glycemic level influence the superiority of successful revascularization?

In the present study, we observed that neither collateral circulation nor syntax score affected the superiority of CTO-SR, and neither did HbA1c.

In terms of collateral circulation, our results were consistent with those from a previous study [41], which enrolled CTO patients with well-developed collateral circulation and demonstrated that revascularization reduced the incidence of cardiac death. Mechanically, although good collateral circulation might offer blood to the occluded area of the RCA, the relationship between collaterals and clinical events remains unclear [30, 42, 43]. Additionally, low diastolic blood pressure [44] and coronary steel [45] can also reduce the protective effects of collaterals. Furthermore, FFR studies [46, 47] observed that collateral circulation affected the evaluation of donor vessel FFR and, hence, the optimal treatment strategy, which might lead to adverse cardiac events. Thus, reperfusion of the RCA-CTO will surely reduce myocardial ischemia as well as cardiac events. On the other hand, diabetic patients were often associated with impaired collateral circulation [23], which makes this reperfusion more essential to perform. In summary, our findings will correct the plausible notion that SR and medical therapy are both acceptable treatments for RCA-CTO patients with well-developed collateral circulation.

When considering coronary severity, we demonstrated that syntax score was a predictive risk factor for mortality. However, the syntax score did not influence the protective effect of CTO-SR. Even though for patients with single-vessel disease, CTO-SR might not offer survival benefit. However, only 209 patients were included in this subgroup, and mortality only occurred in eight patients (cardiac death: four patients), which reduced the strength of the evidence. Further studies that focus on only a single-vessel RCA-CTO in diabetic patients are needed to verify our results.

Some experts observed a connection between glycemic control and clinical outcomes [48]. However, in the present study, we observed that the HbA1c level did not predict mortality. We speculated that the reason might be that the CTO-SR procedure is the main beneficial factor for patients with RCA-CTO. Further studies are still needed to confirm the benefits of glycemic control.

## Limitations

The following limitations were present in this study. (1) This study was a retrospective cohort study. Some baseline variables were imbalanced. Although a propensity score-matched analysis was performed to balance any potential bias, the evidence grade is lower than that of a randomized controlled trial. (2) In the present study, cine angiograms could only be obtained from 794 (84.10%) patients. In the multivariable Cox regression analysis, we included syntax scores, which may omit some adverse cardiac events. (3) For the certifications of the clinical endpoints, TVR and repeat nonfatal MI were difficult to confirm. To ensure accuracy, only patients who had records of rehospitalization in the Beijing Anzhen Hospital or who could provide written certificates of diagnosis (by WeChat) were considered as meeting the definitions of TVR or repeat nonfatal MI. This consideration will introduce some bias. (4) We did not evaluate signs of a viable myocardium.

## Conclusions

In summary, for stable RCA-CTO patients with diabetes, successful revascularization offered patients more clinical benefits than medical therapy. CTO-CABG might be a more recommended way to accomplish revascularization.

## Additional file

**Additional file 1: Table S1.** Baseline Characteristics in Propensity-Matched Population. **Table S2.** Baseline Characteristics in right dominance RCA-CTO. **Table S3.** Clinical Outcomes (Right dominance RCA-CTO). **Table S4.** Baseline Characteristics (initial CTO-MT versus successful CTO-PCI). **Table S5.** Baseline Characteristics (Failed CTO-PCI versus successful CTO-PCI). **Table S6**. Clinical Outcomes (initial CTO-MT versus successful CTO-PCI). **Table S7.** Clinical Outcomes (failed CTO-PCI versus successful CTO-PCI). **Table S8.** Baseline Characteristics (CTO-CABG versus successful CTO-PCI). **Table S9.** Clinical Outcomes (CTO-CABG versus successful CTO-PCI). **Table S10.** Baseline Characteristics (Initial CTO-MT versus CTO-CABG). **Table S11.** Clinical Outcomes (Initial CTO-MT versus CTO-CABG). **Table S12.** Baseline Characteristics (Failed CTO-PCI versus CTO-CABG). **Table S13.** Clinical Outcomes (Failed CTO-PCI versus CTO-CABG). **Figure S1.** Absolute Standard Difference before and after Propensity-Score-Matching.

## Data Availability

The datasets generated and analyzed for this current study are available from the corresponding author upon reasonable request.

## Abbreviations

CTO: chronic total occlusion
PCI: percutaneous transluminal coronary intervention
MT: medical therapy
MACE: major adverse cardiac event
MI: myocardial infarction
TVR: target vessel revascularization
LAD: left anterior descending coronary artery
LCX: left circumflex artery
RCA: right coronary artery
TIMI: thrombolysis in myocardial infarction
AF: atrial fibrillation
CKD: chronic kidney disease
COPD: chronic obstructive pulmonary disease
LVEF: left ventricular ejection fraction
BMI: body mass index
CCB: calcium-channel blocker
ACEI/ARB: angiotensin converting enzyme inhibitor/angiotensin-receptor blocker
HR: hazard ratio
CI: conference interval

## References

[1] Fefer P, Knudtson ML, Cheema AN, Galbraith PD, Osherov AB, Yalonetsky S (2012). Current perspectives on coronary chronic total occlusions: the Canadian Multicenter Chronic Total Occlusions Registry. J Am Coll Cardiol.

[2] Tsai TT, Stanislawski MA, Shunk KA, Armstrong EJ, Grunwald GK, Schob AH (2017). Contemporary incidence, management, and long-term outcomes of percutaneous coronary interventions for chronic coronary artery total occlusions: insights from the VA CART program. JACC Cardiovasc Interv.

[3] Jeroudi OM, Alomar ME, Michael TT, El Sabbagh A, Patel VG, Mogabgab O (2014). Prevalence and management of coronary chronic total occlusions in a tertiary Veterans Affairs hospital. Catheter Cardiovasc Interv.

[4] Werner GS, Gitt AK, Zeymer U, Juenger C, Towae F, Wienbergen H (2009). Chronic total coronary occlusions in patients with stable angina pectoris: impact on therapy and outcome in present day clinical practice. Clin Res Cardiol.

[5] Safley DM, House JA, Marso SP, Grantham JA, Rutherford BD (2008). Improvement in survival following successful percutaneous coronary intervention of coronary chronic total occlusions: variability by target vessel. JACC Cardiovasc Interv.

[6] Mitomo S, Naganuma T, Jabbour RJ, Sato K, Takebayashi H, Kobayashi T (2017). Impact of target vessel on long-term cardiac mortality after successful chronic total occlusion percutaneous coronary intervention: insights from a Japanese multicenter registry. Int J Cardiol.

[7] Bryniarski L, Klima L, Surowiec S, Bryniarski KL, Terlecki M, Dudek D (2018). Does the effectiveness of recanalization of chronic occlusion depend on the location of the obstruction?. Adv Interv Cardiol.

[8] Tajti P, Karmpaliotis D, Alaswad K, Jaffer FA, Yeh RW, Patel M (2019). In-hospital outcomes of chronic total occlusion percutaneous coronary interventions in patients with prior coronary artery bypass graft surgery. Circ Cardiovasc Interv.

[9] Christakopoulos GE, Christopoulos G, Carlino M, Jeroudi OM, Roesle M, Rangan BV (2015). Meta-analysis of clinical outcomes of patients who underwent percutaneous coronary interventions for chronic total occlusions. Am J Cardiol.

[10] Migliorini A, Valenti R, Parodi G, Buonamici P, Cerisano G, Carrabba N (2011). The impact of right coronary artery chronic total occlusion on clinical outcome of patients undergoing percutaneous coronary intervention for unprotected left main disease. J Am Coll Cardiol.

[11] Claessen BE, Dangas GD, Godino C, Henriques JP, Leon MB, Park SJ (2013). Impact of target vessel on long-term survival after percutaneous coronary intervention for chronic total occlusions. Catheter Cardiovasc Interv.

[12] Salisbury AC, Sapontis J, Grantham JA, Qintar M, Gosch KL, Lombardi W (2017). Outcomes of chronic total occlusion percutaneous coronary intervention in patients with diabetes: insights from the OPEN CTO registry. JACC Cardiovasc Interv.

[13] Choi KH, Yang JH, Song YB, Hahn JY, Choi JH, Gwon HC (2017). Long-term clinical outcomes of patients with coronary chronic total occlusion treated with percutaneous coronary intervention versus medical therapy according to presence of diabetes mellitus. EuroIntervention.

[14] Vedantham S, Kluever AK, Deindl E (2018). Is there a chance to promote arteriogenesis by DPP4 inhibitors even in type 2 diabetes? A critical review. Cells.

[15] Guandalini GS, Bangalore S (2018). The potential effects of new stent platforms for coronary revascularization in patients with diabetes. Can J Cardiol.

[16] Overgaard CB, Dzavik V, Buller CE, Liu L, Banasiak W, Devlin G (2013). Percutaneous revascularization and long term clinical outcomes of diabetic patients randomized in the Occluded Artery Trial (OAT). Int J Cardiol.

[17] Neumann FJ, Sousa-Uva M, Ahlsson A, Alfonso F, Banning AP, Benedetto U (2019). 2018 ESC/EACTS Guidelines on myocardial revascularization. Eur Heart J.

[18] Mashaly A, Rha SW, Choi BG, Baek MJ, Ryu YG, Choi SY (2018). Impact of diabetes mellitus on 5-year clinical outcomes in patients with chronic total occlusion lesions. Coron Artery Dis.

[19] Sanguineti F, Garot P, O’Connor S, Watanabe Y, Spaziano M, Lefevre T (2017). Chronic total coronary occlusion treated by percutaneous coronary intervention: long-term outcome in patients with and without diabetes. EuroIntervention.

[20] Sapontis J, Salisbury AC, Yeh RW, Cohen DJ, Hirai T, Lombardi W (2017). Early procedural and health status outcomes after chronic total occlusion angioplasty: a report from the OPEN-CTO Registry (Outcomes, Patient Health Status, and Efficiency in Chronic Total Occlusion Hybrid Procedures). JACC Cardiovasc Interv.

[21] Stefanini GG, Kalesan B, Serruys PW, Heg D, Buszman P, Linke A (2011). Long-term clinical outcomes of biodegradable polymer biolimus-eluting stents versus durable polymer sirolimus-eluting stents in patients with coronary artery disease (LEADERS): 4 year follow-up of a randomised non-inferiority trial. Lancet.

[22] Stone GW, Kandzari DE, Mehran R, Colombo A, Schwartz RS, Bailey S (2005). Percutaneous recanalization of chronically occluded coronary arteries: a consensus document: part I. Circulation.

[23] Shen Y, Ding FH, Dai Y, Wang XQ, Zhang RY, Lu L (2018). Reduced coronary collateralization in type 2 diabetic patients with chronic total occlusion. Cardiovasc Diabetol.

[24] Ponikowski P, Voors AA, Anker SD, Bueno H, Cleland JG, Coats AJ (2016). 2016 ESC Guidelines for the diagnosis and treatment of acute and chronic heart failure: the Task Force for the diagnosis and treatment of acute and chronic heart failure of the European Society of Cardiology (ESC) Developed with the special contribution of the Heart Failure Association (HFA) of the ESC. Eur J Heart Fail.

[25] Cutlip DE, Windecker S, Mehran R, Boam A, Cohen DJ, van Es GA (2007). Clinical end points in coronary stent trials: a case for standardized definitions. Circulation.

[26] Thygesen K, Alpert JS, Jaffe AS, Simoons ML, Chaitman BR, White HD (2012). Third universal definition of myocardial infarction. J Am Coll Cardiol.

[27] Hasegawa T, Godino C, Basavarajaiah S, Takagi K, Rezq A, Latib A (2014). Differences in the clinical and angiographic characteristics of chronic total occlusion lesions in the three major coronary arteries. J Interv Cardiol.

[28] Konstanty-Kalandyk J, Bartus K, Piatek J, Kedziora A, Darocha T, Bryniarski KL (2018). Is right coronary artery chronic total vessel occlusion impacting the surgical revascularization results of patients with multivessel disease? A retrospective study. PeerJ.

[29] Damluji AA, Pomenti SF, Ramireddy A, Al-Damluji MS, Alfonso CE, Schob AH (2016). Influence of total coronary occlusion on clinical outcomes (from the Bypass Angioplasty Revascularization Investigation 2 Diabete sTrial). Am J Cardiol.

[30] Yang ZK, Shen Y, Hu J, Zhang Q, Ding FH, Zhang RY (2017). Impact of coronary collateral circulation on angiographic in-stent restenosis in patients with stable coronary artery disease and chronic total occlusion. Int J Cardiol.

[31] Kennedy MW, Fabris E, Suryapranata H, Kedhi E (2017). Is ischemia the only factor predicting cardiovascular outcomes in all diabetes mellitus patients?. Cardiovasc Diabetol.

[32] Orbach A, Halon DA, Jaffe R, Rubinshtein R, Karkabi B, Flugelman MY (2018). Impact of diabetes and early revascularization on the need for late and repeat procedures. Cardiovasc Diabetol.

[33] Kogan A, Ram E, Levin S, Fisman EZ, Tenenbaum A, Raanani E (2018). Impact of type 2 diabetes mellitus on short- and long-term mortality after coronary artery bypass surgery. Cardiovasc Diabetol.

[34] Zhang HP, Ai H, Zhao Y, Li H, Tang GD, Zheng NX (2018). Effect of chronic total occlusion percutaneous coronary intervention on clinical outcomes in elderly patients. Am J Med Sci.

[35] Gebhard C, Gick M, Ferenc M, Stahli BE, Ademaj F, Mashayekhi K (2018). Coronary dominance and prognosis in patients with chronic total occlusion treated with percutaneous coronary intervention. Catheter Cardiovasc Interv.

[36] Schoenenberger AW, Jamshidi P, Kobza R, Zuber M, Stuck AE, Pfisterer M (2010). Progression of coronary artery disease during long-term follow-up of the Swiss Interventional Study on Silent Ischemia Type II (SWISSI II). Clin Cardiol.

[37] Jimenez-Quevedo P, Sabate M, Angiolillo DJ, Alfonso F, Hernandez-Antolin R, SanMartin M (2007). Long-term clinical benefit of sirolimus-eluting stent implantation in diabetic patients with de novo coronary stenoses: long-term results of the DIABETES trial. Eur Heart J.

[38] Loutfi M, Mulvihill NT, Boccalatte M, Farah B, Fajadet J, Marco J (2003). Impact of restenosis and disease progression on clinical outcome after multivessel stenting in diabetic patients. Catheter Cardiovasc Interv.

[39] Claessen BE, Hoebers LP, van der Schaaf RJ, Kikkert WJ, Engstrom AE, Vis MM (2010). Prevalence and impact of a chronic total occlusion in a non-infarct-related artery on long-term mortality in diabetic patients with ST elevation myocardial infarction. Heart.

[40] Nombela-Franco L, Mitroi CD, Fernandez-Lozano I, Garcia-Touchard A, Toquero J, Castro-Urda V (2012). Ventricular arrhythmias among implantable cardioverter-defibrillator recipients for primary prevention: impact of chronic total coronary occlusion (VACTO Primary Study). Circ Arrhythm Electrophysiol.

[41] Jang WJ, Yang JH, Choi SH, Song YB, Hahn JY, Choi JH (2015). Long-term survival benefit of revascularization compared with medical therapy in patients with coronary chronic total occlusion and well-developed collateral circulation. JACC Cardiovasc Interv.

[42] Wright S, Lichtenstein M, Grigg L, Sivaratnam D (2013). Myocardial perfusion imaging (MPI) is superior to the demonstration of distal collaterals in predicting cardiac events in chronic total occlusion (CTO). J Nucl Cardiol.

[43] Aboul-Enein F, Kar S, Hayes SW, Sciammarella M, Abidov A, Makkar R (2004). Influence of angiographic collateral circulation on myocardial perfusion in patients with chronic total occlusion of a single coronary artery and no prior myocardial infarction. J Nucl Med.

[44] Shen Y, Yang ZK, Hu J, Wang XQ, Dai Y, Zhang S (2018). Donor artery stenosis interactions with diastolic blood pressure on coronary collateral flow in type 2 diabetic patients with chronic total occlusion. Cardiovasc Diabetol.

[45] Werner GS, Fritzenwanger M, Prochnau D, Schwarz G, Ferrari M, Aarnoudse W (2006). Determinants of coronary steal in chronic total coronary occlusions donor artery, collateral, and microvascular resistance. J Am Coll Cardiol.

[46] Ladwiniec A, Cunnington MS, Rossington J, Mather AN, Alahmar A, Oliver RM (2015). Collateral donor artery physiology and the influence of a chronic total occlusion on fractional flow reserve. Circ Cardiovasc Interv.

[47] Ladwiniec A, Hoye A (2015). The haemodynamic effects of collateral donation to a chronic total occlusion: implications for patient management. Int J Cardiol.

[48] Hwang JK, Lee SH, Song YB, Ahn J, Carriere K, Jang MJ (2017). Glycemic control status after percutaneous coronary intervention and long-term clinical outcomes in patients with type 2 diabetes mellitus. Circ Cardiovasc Interv.

