# Supplementary material for: Successful revascularization versus medical therapy in diabetic patients with stable right coronary artery chronic total occlusion: a retrospective cohort study
Source: Cardiovasc Diabetol. 2019 Aug 21;18:108. doi: 10.1186/s12933-019-0911-4 (PMC6702731; doi:10.1186/s12933-019-0911-4)
Supplement: Supplementary file 1 — Additional file 1: Table S1. Baseline Characteristics in Propensity-Matched Population. Table S2. Baseline Characteristics in right dominance RCA-CTO. Table S3. Clinical Outcomes (Right dominance RCA-CTO). Table S4. Baseline Characteristics (initial CTO-MT versus successful CTO-PCI). Table S5. Baseline Characteristics (Failed CTO-PCI versus successful CTO-PCI). Table S6. Clinical Outcomes (initial CTO-MT versus successful CTO-PCI). Table S7. Clinical Outcomes (failed CTO-PCI versus successful CTO-PCI). Table S8. Baseline Characteristics (CTO-CABG versus successful CTO-PCI). Table S9. Clinical Outcomes (CTO-CABG versus successful CTO-PCI). Table S10. Baseline Characteristics (Initial CTO-MT versus CTO-CABG). Table S11. Clinical Outcomes (Initial CTO-MT versus CTO-CABG). Table S12. Baseline Characteristics (Failed CTO-PCI versus CTO-CABG). Table S13. Clinical Outcomes (Failed CTO-PCI versus CTO-CABG). Figure S1. Absolute Standard Difference before and after Propensity-Score-Matching. [file 12933_2019_911_MOESM1_ESM.docx]

**(1), Table S1 Baseline Characteristics in Propensity-Matched Population**

**(2), Table S2 Baseline Characteristics in right dominance RCA-CTO**

**(3), Table S3 Clinical Outcomes (Right dominance RCA-CTO)**

**(4), Table S4 Baseline Characteristics (initial CTO-MT versus successful CTO-PCI)**

**(5), Table S5 Baseline Characteristics (Failed CTO-PCI versus successful CTO-PCI)**

**(6), Table S6 Clinical Outcomes (initial CTO-MT versus successful CTO-PCI)**

**(7), Table S7 Clinical Outcomes (failed CTO-PCI versus successful CTO-PCI)**

**(8), Table S8 Baseline Characteristics (CTO-CABG versus successful CTO-PCI)**

**(9), Table S9 Clinical Outcomes (CTO-CABG versus successful CTO-PCI)**

**(10), Table S10 Baseline Characteristics (Initial CTO-MT versus CTO-CABG)**

**(11), Table S11 Clinical Outcomes (Initial CTO-MT versus CTO-CABG)**

**(12), Table S12 Baseline Characteristics (Failed CTO-PCI versus CTO-CABG)**

**(13), Table S13 Clinical Outcomes (Failed CTO-PCI versus CTO-CABG)**

**(14), Figure S1 Absolute Standard Difference before and after** **Propensity-Score-Matching**

| **Table S1 Baseline Characteristics in Propensity Matched Population (n=572)** | | | |
| --- | --- | --- | --- |
|  | Initial CTO-MT (n=286) | Successful CTO-PCI (n=286) | P value |
| **Clinical Characteristics** | | | |
| Age (yrs) | 60.00±10.84 | 60.49±8.61 | 0.550 |
| Male | 214(74.8) | 214(74.8) | 1.000 |
| Hypertension | 189(66.1) | 191(66.8) | 0.859 |
| Dyslipidemia | 96(33.6) | 89(31.1) | 0.532 |
| PVD | 11(3.8) | 13(4.5) | 0.677 |
| Prior MI | 166(58.0) | 154(53.8) | 0.312 |
| Prior PCI | 51(17.8) | 48(16.8) | 0.740 |
| Prior stroke | 17(5.9) | 16(5.6) | 0.858 |
| **Heart failure** | 94(32.9) | 90(31.5) | 0.720 |
| Systolic heart failure | 41(14.3) | 40(14.0) | 0.905 |
| Diastolic heart failure | 53(18.5) | 50(17.5) | 0.744 |
| CKD | 8(2.8) | 7(2.4) | 0.794 |
| COPD /asthma | 1(0.3) | 2(0.7) | 1.000 |
| Hyperuricemia | 63(22.0) | 60(21.0) | 0.760 |
| Smoking | 146(51.0) | 147(51.4) | 0.933 |
| Drinking | 45(15.7) | 57(19.9) | 0.190 |
| BMI (kg/m^2^) | 26.43(24.45-28.56) | 26.48(24.47-28.04) | 0.582 |
| LVEF (%) | 61.00(54.00-66.00) | 60.00(54.50-66.00) | 0.649 |
| RWMA | 100(35.0) | 92(32.2) | 0.479 |
| Fasting blood glucose(mmol/L) | 7.10(6.34-8.87) | 7.32(6.15-9.09) | 0.828 |
| HbA1c(%) | 7.30(6.70-7.90) | 7.30(6.80-8.10) | 0.930 |
| **Medical Treatment** | | | |
| Aspirin | 278(97.2) | 279(97.6) | 0.794 |
| P2Y_12_ inhibitor | 253(88.5) | 253(88.5) | 1.000 |
| Statin | 271(94.8) | 272(95.1) | 0.849 |
| Nitrites | 132(46.2) | 128(44.8) | 0.737 |
| Beta-blocker | 225(78.7) | 218(76.2) | 0.484 |
| CCB | 74(25.9) | 66(23.1) | 0.437 |
| ACEI/ARB | 158(55.2) | 144(50.3) | 0.241 |
| Insulin | 102(35.7) | 99(34.6) | 0.793 |
| Sulfonylureas | 40(14.0) | 40(14.0) | 1.000 |
| Glinide | 9(3.1) | 13(4.5) | 0.384 |
| Biguanides | 101(35.3) | 113(39.5) | 0.300 |
| Thiazolidinediones | 31(10.8) | 30(10.5) | 0.892 |
| Alpha-glucosidase inhibitor | 98(34.3) | 89(31.1) | 0.422 |
| **Angiographic Characteristics** | | | |
| Dominance artery (right) | 276(96.5) | 276(96.5) | 1.000 |
| **Number of Diseased Vessels** | | | |
| 1 | 63(22.0) | 66(23.1) | 0.764 |
| 2 | 113(39.5) | 109(38.1) | 0.731 |
| 3 | 110(38.5) | 111(38.8) | 0.932 |
| Syntax score | 20.00(15.00-27.00) | 20.00(14.00-23.00) | 0.665 |
| Rentrop grade≥2 | 205(84.4) | 212(85.8) | 0.648 |
| Abrupt stump | 123(50.6) | 128(51.8) | 0.790 |
| Calcification | 45(18.5) | 46(18.6) | 0.976 |
| Bending ≥45° | 210(86.4) | 215(87.0) | 0.838 |
| CTO length≥20mm | 175(72.0) | 187(75.7) | 0.352 |
| Values are n(%), mean±SD or median with interquartile range.  PCI: percutaneous transluminal coronary intervention; MT: medical therapy; CABG: coronary artery bypass grafting; PVD: peripheral vascular disease; MI: myocardial infarction; CKD: Chronic kidney disease; COPD: Chronic obstructive pulmonary disease; LVEF: left ventricular ejection fraction; BMI: body mass index; CCB: calcium-channel blocker; ACEI/ARB: angiotensin converting enzyme inhibitor/angiotensin-receptor blocker; CTO: chronic total occlusion; HF: heart failure; RWMA: reginal wall motion abnormality. | | | |

| **Table S2 Baseline Characteristics in right dominance RCA-CTO (n=884)** | | | |
| --- | --- | --- | --- |
|  | CTO-MT (n=396) | CTO-SR(n=488) | P value |
| **Clinical Characteristics** | | | |
| Age (yrs) | 60.55±10.74 | 59.98±8.78 | 0.393 |
| Male | 292(73.7) | 385(78.9) | 0.072 |
| Hypertension | 275(69.4) | 328(67.2) | 0.479 |
| Dyslipidemia | 141(35.6) | 137(28.1) | **0.016** |
| PVD | 16(4.0) | 14(2.9) | 0.339 |
| Prior MI | 210(53.0) | 278(57.0) | 0.242 |
| Prior PCI | 84(21.2) | 71(14.5) | **0.010** |
| Prior stroke | 30(7.6) | 21(4.3) | **0.038** |
| **Heart failure** | 115(29.0) | 147(30.1) | 0.726 |
| Systolic heart failure | 48(12.1) | 62(12.7) | 0.794 |
| Diastolic heart failure | 67(16.9) | 85(17.4) | 0.845 |
| CKD | 15(3.8) | 9(1.8) | 0.077 |
| COPD /asthma | 4(1.0) | 4(0.8) | 1.000 |
| Hyperuricemia | 89(22.5) | 85(17.4) | 0.060 |
| Smoking | 197(49.7) | 259(53.1) | 0.325 |
| Drinking | 68(17.2) | 90(18.4) | 0.624 |
| BMI (kg/m^2^) | 26.48(24.44-28.44) | 26.48(24.70-28.37) | 0.483 |
| LVEF (%) | 61.00(55.00-66.00) | 60.00(55.00-66.00) | 0.269 |
| RWMA | 133(33.6) | 156(32.0) | 0.610 |
| Fasting blood glucose(mmol/L) | 7.22(6.37-9.21) | 7.56(6.25-9.39) | 0.487 |
| HbA1c(%)^ψ^ | 7.30(6.70-8.13) | 7.30(6.80-8.10) | 0.857 |
| **Medical Treatment** | | | |
| Aspirin | 384(97.0) | 478(98.4) | 0.170 |
| P2Y_12_ inhibitor | 359(90.7) | 403(82.9) | **0.001** |
| Statin | 380(96.0) | 451(92.8) | **0.045** |
| Nitrites | 240(60.6) | 152(31.3) | **0.000** |
| Beta-blocker | 304(76.8) | 398(81.9) | 0.060 |
| CCB | 114(28.8) | 119(24.5) | 0.149 |
| ACEI/ARB | 225(56.8) | 267(54.9) | 0.576 |
| Insulin | 145(36.6) | 199(40.8) | 0.207 |
| Sulfonylureas | 57(14.4) | 66(13.5) | 0.710 |
| Glinide | 16(4.0) | 17(3.5) | 0.664 |
| Biguanides | 133(33.6) | 189(38.7) | 0.114 |
| Thiazolidinediones | 43(10.9) | 52(10.7) | 0.923 |
| Alpha-glucosidase inhibitor | 143(36.1) | 146(29.9) | 0.051 |
| **Number of Diseased Vessels** | | | |
| 1 | 107(27.0) | 94(19.3) | **0.006** |
| 2 | 138(34.8) | 196(40.2) | 0.105 |
| 3 | 151(38.1) | 198(40.6) | 0.460 |
| Syntax score^#^ | 20.00(13.00-27.00) | 20.00(16.25-23.00) | 0.387 |
| Rentrop grade≥2^#^ | 292(84.6) | 367(88.6) | 0.104 |
| Abrupt stump^#^ | 172(49.9) | 204(49.3) | 0.874 |
| Calcification^#^ | 69(20.0) | 80(19.3) | 0.815 |
| Bending ≥45°^#^ | 300(87.0) | 357(86.2) | 0.771 |
| CTO length≥20mm^#^ | 256(74.2) | 304(73.4) | 0.809 |
| **Procedural Characteristics** |  |  |  |
| Retrograde approach^*^ | 3(1.6) | 40(13.1) | **0.000** |
| Values are n(%), mean±SD or median with interquartile range.  PCI: percutaneous transluminal coronary intervention; MT: medical therapy; CABG: coronary artery bypass grafting; PVD: peripheral vascular disease; MI: myocardial infarction; CKD: Chronic kidney disease; COPD: Chronic obstructive pulmonary disease; LVEF: left ventricular ejection fraction; BMI: body mass index; CCB: calcium-channel blocker; ACEI/ARB: angiotensin converting enzyme inhibitor/angiotensin-receptor blocker; CTO: chronic total occlusion; HF: heart failure; RWMA: reginal wall motion abnormality.  ^#^Cine angiograms records got from 759 (85.86%) individuals.  ^ψ^HbA1c got from 840 (95.00%) individuals.  ^*^ Only patients who were treated with PCI. | | | |

| **Table S3 Clinical Outcomes (Right dominance RCA-CTO)** | | | |
| --- | --- | --- | --- |
|  | CTO-MT | CTO-SR | P value |
| **All cause death** |  |  |  |
| Event per 1000 patient-years | 29.59 | 12.48 | **0.000** |
| Unadjusted HR (95% CI) | 1 | **0.413(0.262-0.651)** | **0.000** |
| Adjusted HR (95% CI) | 1 | **0.460(0.284-0.743)** | **0.001** |
| **Non-cardiac death** |  |  |  |
| Event per 1000 patient-years | 6.64 | 5.41 | 0.614 |
| Unadjusted HR (95% CI) | 1 | 0.742(0.331-1.664) | 0.469 |
| Adjusted HR (95% CI) | 1 | 1.731(0.663-4.518) | 0.262 |
| **Cardiac death** |  |  |  |
| Event per 1000 patient-years | 22.95 | 7.07 | **0.000** |
| Unadjusted HR (95% CI) | 1 | **0.312(0.176-0.553)** | **0.000** |
| Adjusted HR (95% CI) | 1 | **0.324(0.177-0.593)** | **0.000** |
| **Probable/Definite cardiac death** |  |  |  |
| Event per 1000 patient-years | 12.08 | 3.73 | **0.002** |
| Unadjusted HR (95% CI) | 1 | **0.310(0.141-0.684)** | **0.004** |
| Adjusted HR (95% CI) | 1 | **0.391(0.168-0.908)** | **0.029** |
| **Repeat nonfatal MI** |  |  |  |
| Event per 1000 patient-years | 15.56 | 6.74 | **0.007** |
| Unadjusted HR (95% CI) | 1 | **0.405(0.216-0.762)** | **0.005** |
| Adjusted HR (95% CI) | 1 | **0.440(0.222-0.872)** | **0.019** |
| **Repeat revascularization** |  |  |  |
| Event per 1000 patient-years | 66.00 | 38.93 | **0.000** |
| Unadjusted HR (95% CI) | 1 | **0.608(0.453-0.816)** | **0.001** |
| Adjusted HR (95% CI) | 1 | **0.626(0.460-0.854)** | **0.003** |
| **TVR** |  |  |  |
| Event per 1000 patient-years | 41.61 | 21.03 | **0.000** |
| Unadjusted HR (95% CI) | 1 | **0.531(0.364-0.774)** | **0.001** |
| Adjusted HR (95% CI) | 1 | **0.546(0.367-0.811)** | **0.003** |
| Adjusted covariates: age, sex, PVD, CKD, COPD, prior MI, systolic HF, LVEF, reginal wall motion abnormality, single vessel disease, triple-vessel disease, syntax scores and HbA1c.  HR: hazard ratio; CI: conference interval; other abbreviations as in **Table 1**. | | | |

| **Table S4 Baseline Characteristics (****initial CTO-MT versus successful CTO-PCI)** | | | |
| --- | --- | --- | --- |
|  | Initial CTO-MT (n=233) | Successful CTO-PCI (n=309) | P value |
| **Clinical Characteristics** | | | |
| Age (yrs) | 62.19±10.08 | 59.06±9.03 | **0.000** |
| Male | 160(68.7) | 245(79.3) | **0.005** |
| Hypertension | 155(66.5) | 206(66.7) | 0.972 |
| Dyslipidemia | 78(33.5) | 102(33.0) | 0.909 |
| PVD | 9(3.9) | 11(3.6) | 0.853 |
| Prior MI | 127(54.5) | 164(53.1) | 0.741 |
| Prior PCI | 49(21.0) | 56(18.1) | 0.397 |
| Prior stroke | 19(8.2) | 7(2.3) | **0.001** |
| **Heart failure** | 74(31.8) | 69(22.3) | **0.014** |
| Systolic heart failure | 38(16.3) | 23(7.4) | **0.001** |
| Diastolic heart failure | 36(15.5) | 46(14.9) | 0.856 |
| CKD | 13(5.6) | 3(1.0) | **0.002** |
| COPD /asthma | 2(0.9) | 2(0.6) | 1.000 |
| Hyperuricemia | 56(24.0) | 48(15.5) | **0.013** |
| Smoking | 112(48.1) | 171(55.3) | 0.093 |
| Drinking | 38(16.3) | 55(17.8) | 0.649 |
| BMI (kg/m^2^) | 26.12(24.07-28.35) | 26.48(24.63-28.41) | 0.094 |
| LVEF (%) | 61.00(53.00-66.00) | 62.00(57.00-66.00) | 0.342 |
| RWMA | 79(33.9) | 74(23.9) | **0.011** |
| Fasting blood glucose(mmol/L) | 7.12(6.23-9.12) | 7.41(6.15-9.22) | 0.636 |
| HbA1c(%) | 7.30(6.80-8.00) | 7.30(6.80-8.10) | 0.823 |
| **Medical Treatment** | | | |
| Aspirin | 227(97.4) | 308(99.7) | **0.046** |
| P2Y_12_ inhibitor | 206(88.4) | 304(98.4) | **0.000** |
| Statin | 226(97.0) | 301(97.4) | 0.770 |
| Nitrites | 120(51.5) | 74(23.9) | **0.000** |
| Beta-blocker | 174(74.7) | 244(79.0) | 0.240 |
| CCB | 67(28.8) | 73(23.6) | 0.177 |
| ACEI/ARB | 136(58.4) | 170(55.0) | 0.436 |
| Insulin | 82(35.2) | 134(43.4) | 0.054 |
| Sulfonylureas | 33(14.2) | 35(11.3) | 0.324 |
| Glinide | 8(3.4) | 11(3.6) | 0.937 |
| Biguanides | 79(33.9) | 123(39.8) | 0.160 |
| Thiazolidinediones | 21(9.0) | 31(10.0) | 0.690 |
| Alpha-glucosidase inhibitor | 87(37.3) | 83(26.9) | 0.009 |
| **Angiographic Characteristics** | | | |
| Dominance artery (right) | 214(91.8) | 305(98.7) | **0.000** |
| **Number of Diseased Vessels** | | | |
| 1 | 48(20.6) | 89(28.8) | **0.030** |
| 2 | 80(34.3) | 140(45.3) | **0.010** |
| 3 | 105(45.1) | 80(25.9) | **0.000** |
| Syntax score | 20.00(17.00-27.00) | 19.00(12.00-21.00) | **0.000** |
| Rentrop grade≥2 | 168(85.7) | 249(91.5) | **0.046** |
| Abrupt stump | 111(56.6) | 136(50.0) | 0.156 |
| Calcification | 40(20.4) | 45(16.5) | 0.285 |
| Bending ≥45° | 170(86.7) | 232(85.3) | 0.659 |
| CTO length≥20mm | 146(74.5) | 191(70.2) | 0.310 |
| Values are n(%), mean±SD or median with interquartile range.  PCI: percutaneous transluminal coronary intervention; MT: medical therapy; CABG: coronary artery bypass grafting; PVD: peripheral vascular disease; MI: myocardial infarction; CKD: Chronic kidney disease; COPD: Chronic obstructive pulmonary disease; LVEF: left ventricular ejection fraction; BMI: body mass index; CCB: calcium-channel blocker; ACEI/ARB: angiotensin converting enzyme inhibitor/angiotensin-receptor blocker; CTO: chronic total occlusion; HF: heart failure; RWMA: reginal wall motion abnormality. | | | |

| **Table S5 Baseline Characteristics (Failed CTO-PCI versus successful CTO-PCI)** | | | |
| --- | --- | --- | --- |
|  | Failed CTO-PCI (n=191) | Successful CTO-PCI (n=309) | P value |
| **Clinical Characteristics** | | | |
| Age (yrs) | 58.73±11.09 | 59.06±9.03 | 0.726 |
| Male | 151(79.1) | 245(79.3) | 0.951 |
| Hypertension | 138(72.3) | 206(66.7) | 0.190 |
| Dyslipidemia | 72(37.7) | 102(33.0) | 0.285 |
| PVD | 7(3.7) | 11(3.6) | 0.951 |
| Prior MI | 97(50.8) | 164(53.1) | 0.619 |
| Prior PCI | 41(21.5) | 56(18.1) | 0.368 |
| Prior stroke | 14(7.3) | 7(2.3) | **0.006** |
| **Heart failure** | 47(24.6) | 69(22.3) | 0.558 |
| Systolic heart failure | 11(5.8) | 23(7.4) | 0.467 |
| Diastolic heart failure | 36(18.8) | 46(14.9) | 0.245 |
| CKD | 2(1.0) | 3(1.0) | 1.000 |
| COPD /asthma | 2(1.0) | 2(0.6) | 0.639 |
| Hyperuricemia | 39(20.4) | 48(15.5) | 0.162 |
| Smoking | 96(50.3) | 171(55.3) | 0.269 |
| Drinking | 33(17.3) | 55(17.8) | 0.882 |
| BMI (kg/m^2^) | 26.84(24.95-28.71) | 26.48(24.63-28.41) | 0.751 |
| LVEF (%) | 62.00(56.00-66.00) | 62.00(57.00-66.00) | 0.762 |
| RWMA | 57(29.8) | 74(23.9) | 0.145 |
| Fasting blood glucose(mmol/L) | 7.30(6.32-9.39) | 7.41(6.15-9.22) | 0.922 |
| HbA1c(%) | 7.30(6.60-8.30) | 7.30(6.80-8.10) | 0.585 |
| **Medical Treatment** | | | |
| Aspirin | 184(96,3) | 308(99.7) | **0.004** |
| P2Y_12_ inhibitor | 179(93.7) | 304(98.4) | **0.005** |
| Statin | 181(94.8) | 301(97.4) | 0.123 |
| Nitrites | 136(71.2) | 74(23.9) | **0.000** |
| Beta-blocker | 153(80.1) | 244(79.0) | 0.759 |
| CCB | 53(27.7) | 73(23.6) | 0.302 |
| ACEI/ARB | 104(54.5) | 170(55.0) | 0.902 |
| Insulin | 71(37.2) | 134(43.4) | 0.171 |
| Sulfonylureas | 28(14.7) | 35(11.3) | 0.275 |
| Glinide | 11(5.8) | 11(3.6) | 0.244 |
| Biguanides | 65(34.0) | 123(39.8) | 0.195 |
| Thiazolidinediones | 26(13.6) | 31(10.0) | 0.221 |
| Alpha-glucosidase inhibitor | 62(32.5) | 83(26.9) | 0.180 |
| **Angiographic Characteristics** | | | |
| Dominance artery (right) | 182(95.3) | 305(98.7) | **0.041** |
| **Number of Diseased Vessels** | | | |
| 1 | 67(35.1) | 89(28.8) | 0.141 |
| 2 | 67(35.1) | 140(45.3) | **0.024** |
| 3 | 57(29.8) | 80(25.9) | 0.336 |
| Syntax score | 19.00(12.00-27.00) | 19.00(12.00-21.00) | 0.361 |
| Rentrop grade≥2 | 129(78.7) | 249(91.5) | **0.000** |
| Abrupt stump | 69(42.1) | 136(50.0) | 0.108 |
| Calcification | 31(18.9) | 45(16.5) | 0.529 |
| Bending ≥45° | 144(87.8) | 232(85.3) | 0.461 |
| CTO length≥20mm | 124(75.6) | 191(70.2) | 0.223 |
| **Procedural Characteristics** |  |  |  |
| Retrograde approach | 3(1.6) | 41(13.3) | **0.000** |
| Perforation^*^ | 4(2.1) | 1(0.3) | 0.073 |
| Pericardial effusion^*^ | 0(0) | 0(0) | **-** |
| Emergency surgery^*^ | 0(0) | 0(0) | **-** |
| Contrast retention/Dissection^*^ | 2(1.0) | 5(1.6) | 0.713 |
| Thread off^*^ | 1(0.5) | 0(0) | 0.382 |
| Sudden cardiac arrest^*^ | 0(0) | 1(0.3) | 1.000 |
| Death during hospitalization^*^ | 0(0) | 0(0) | - |
| Values are n(%), mean±SD or median with interquartile range.  PCI: percutaneous transluminal coronary intervention; MT: medical therapy; CABG: coronary artery bypass grafting; PVD: peripheral vascular disease; MI: myocardial infarction; CKD: Chronic kidney disease; COPD: Chronic obstructive pulmonary disease; LVEF: left ventricular ejection fraction; BMI: body mass index; CCB: calcium-channel blocker; ACEI/ARB: angiotensin converting enzyme inhibitor/angiotensin-receptor blocker; CTO: chronic total occlusion; HF: heart failure; RWMA: reginal wall motion abnormality. | | | |

| **Table S6 Clinical Outcomes (initial CTO-MT versus successful CTO-PCI)** | | | |
| --- | --- | --- | --- |
|  | Initial CTO-MT | Successful CTO-PCI | P value |
| **All cause death** |  |  |  |
| Event per 1000 patient-years | 32.01 | 10.44 | **0.000** |
| Unadjusted HR (95% CI) | 1 | **0.326(0.176-0.606)** | **0.000** |
| Adjusted HR (95% CI) | 1 | **0.498(0.251-0.987)** | **0.046** |
| **Noncardiac death** |  |  |  |
| Event per 1000 patient-years | 7.23 | 4.18 | 0.316 |
| Unadjusted HR (95% CI) | 1 | 0.594(0.199-1.769) | 0.349 |
| Adjusted HR (95% CI) | 1 | 1.640(0.410-6.564) | 0.484 |
| **Cardiac death** |  |  |  |
| Event per 1000 patient-years | 24.79 | 6.26 | **0.000** |
| Unadjusted HR (95% CI) | 1 | **0.250(0.116-0.540)** | **0.000** |
| Adjusted HR (95% CI) | 1 | **0.371(0.154-0.892)** | **0.027** |
| **Probable/Definite cardiac death** |  |  |  |
| Event per 1000 patient-years | 13.43 | 3.48 | **0.005** |
| Unadjusted HR (95% CI) | 1 | **0.248(0.088-0.702)** | **0.009** |
| Adjusted HR (95% CI) | 1 | 0.463(0.126-1.701) | 0.246 |
| **Repeat nonfatal MI** |  |  |  |
| Event per 1000 patient-years | 16.95 | 9.21 | 0.095 |
| Unadjusted HR (95% CI) | 1 | 0.533(0.256-1.111) | 0.093 |
| Adjusted HR (95% CI) | 1 | 0.639(0.280-1.461) | 0.289 |
| **Repeat revascularization** |  |  |  |
| Event per 1000 patient-years | 47.89 | 59.74 | 0.233 |
| Unadjusted HR (95% CI) | 1 | 1.249(0.858-1.818) | 0.246 |
| Adjusted HR (95% CI) | 1 | 1.113(0.745-1.661) | 0.602 |
| **TVR** |  |  |  |
| Event per 1000 patient-years | 30.49 | 35.20 | 0.539 |
| Unadjusted HR (95% CI) | 1 | 1.141(0.715-1.820) | 0.580 |
| Adjusted HR (95% CI) | 1 | 0.973(0.591-1.602) | 0.914 |
| Adjusted covariates (model 2): age, sex, PVD, CKD, COPD, prior MI, systolic HF, LVEF, reginal wall motion abnormality, single vessel disease, triple-vessel disease, syntax scores and HbA1c.  HR: hazard ratio; CI: conference interval; other abbreviations as in **Table 1**. | | | |

| **Table S7 Clinical Outcomes (failed CTO-PCI versus successful CTO-PCI)** | | | |
| --- | --- | --- | --- |
|  | Failed CTO-PCI | Successful CTO-PCI | P value |
| **All cause death** |  |  |  |
| Event per 1000 patient-years | 25.39 | 10.44 | **0.006** |
| Unadjusted HR (95% CI) | 1 | **0.411(0.212-0.797)** | **0.009** |
| Adjusted HR (95% CI) | 1 | **0.315(0.154-0.642)** | **0.001** |
| **Noncardiac death** |  |  |  |
| Event per 1000 patient-years | 4.84 | 4.18 | 0.819 |
| Unadjusted HR (95% CI) | 1 | 0.830(0.234-2.945) | 0.774 |
| Adjusted HR (95% CI) | 1 | 1.879(0.375-9.413) | 0.443 |
| **Cardiac death** |  |  |  |
| Event per 1000 patient-years | 20.56 | 6.26 | **0.002** |
| Unadjusted HR (95% CI) | 1 | **0.309(0.138-0.694)** | **0.004** |
| Adjusted HR (95% CI) | 1 | **0.195(0.082-0.465)** | **0.000** |
| **Probable/Definite cardiac death** |  |  |  |
| Event per 1000 patient-years | 10.88 | 3.48 | **0.030** |
| Unadjusted HR (95% CI) | 1 | **0.320(0.107-0.955)** | **0.041** |
| Adjusted HR (95% CI) | 1 | **0.151(0.041-0.563)** | **0.005** |
| **Repeat nonfatal MI** |  |  |  |
| Event per 1000 patient-years | 11.21 | 9.21 | 0.647 |
| Unadjusted HR (95% CI) | 1 | 0.812(0.347-1.902) | 0.632 |
| Adjusted HR (95% CI) | 1 | 0.764(0.285-2.049) | 0.592 |
| **Repeat revascularization** |  |  |  |
| Event per 1000 patient-years | 76.80 | 59.74 | 0.150 |
| Unadjusted HR (95% CI) | 1 | 0.779(0.546-1.110) | 0.167 |
| Adjusted HR (95% CI) | 1 | 0.712(0.490-1.033) | 0.074 |
| **TVR** |  |  |  |
| Event per 1000 patient-years | 48.21 | 35.20 | 0.153 |
| Unadjusted HR (95% CI) | 1 | 0.736(0.473-1.145) | 0.174 |
| Adjusted HR (95% CI) | 1 | 0.687(0.432-1.091) | 0.112 |
| Adjusted covariates (model 2): age, sex, PVD, CKD, COPD, prior MI, systolic HF, LVEF, reginal wall motion abnormality, single vessel disease, triple-vessel disease, syntax scores and HbA1c.  HR: hazard ratio; CI: conference interval; other abbreviations as in **Table 1**. | | | |

| **Table S8 Baseline Characteristics (CTO-CABG versus successful CTO-PCI)** | | | |
| --- | --- | --- | --- |
|  | CTO-CABG (n=191) | Successful CTO-PCI (n=309) | P value |
| **Clinical Characteristics** | | | |
| Age (yrs) | 61.43±8.17 | 59.06±9.03 | **0.003** |
| Male | 147(77.0) | 245(79.3) | 0.539 |
| Hypertension | 131(68.6) | 206(66.7) | 0.656 |
| Dyslipidemia | 37(19.4) | 102(33.0) | **0.001** |
| PVD | 4(2.1) | 11(3.6) | 0.351 |
| Prior MI | 121(63.4) | 164(53.1) | **0.024** |
| Prior PCI | 16(8.4) | 56(18.1) | **0.003** |
| Prior stroke | 15(7.9) | 7(2.3) | **0.003** |
| **Heart failure** | 80(41.9) | 69(22.3) | **0.000** |
| Systolic heart failure | 40(20.9) | 23(7.4) | **0.000** |
| Diastolic heart failure | 40(20.9) | 46(14.9) | 0.081 |
| CKD | 7(3.7) | 3(1.0) | 0.078 |
| COPD /asthma | 2(1.0) | 2(0.6) | 0.639 |
| Hyperuricemia | 38(19.9) | 48(15.5) | 0.209 |
| Smoking | 95(49.7) | 171(55.3) | 0.223 |
| Drinking | 36(18.8) | 55(17.8) | 0.768 |
| BMI (kg/m^2^) | 26.48(24.64-28.33) | 26.48(24.63-28.41) | 0.596 |
| LVEF (%) | 59.00(52.00-64.00) | 62.00(57.00-66.00) | **0.000** |
| RWMA | 86(45.0) | 74(23.9) | **0.000** |
| Fasting blood glucose(mmol/L) | 7.85(6.13-9.37) | 7.41(6.15-9.22) | 0.838 |
| HbA1c(%) | 7.30(7.10-7.43) | 7.30(6.80-8.10) | 0.281 |
| **Medical Treatment** | | | |
| Aspirin | 182(96.3) | 308(99.7) | **0.004** |
| P2Y_12_ inhibitor | 106(56.1) | 304(98.4) | **0.000** |
| Statin | 161(85.2) | 301(97.4) | **0.000** |
| Nitrites | 78(41.3) | 74(23.9) | **0.000** |
| Beta-blocker | 161(85.2) | 244(79.0) | 0.084 |
| CCB | 50(26.5) | 73(23.6) | 0.477 |
| ACEI/ARB | 102(54.0) | 170(55.0) | 0.820 |
| Insulin | 68(35.6) | 134(43.4) | 0.086 |
| Sulfonylureas | 31(16.2) | 35(11.3) | 0.116 |
| Glinide | 7(3.7) | 11(3.6) | 0.951 |
| Biguanides | 72(37.7) | 123(39.8) | 0.638 |
| Thiazolidinediones | 23(12.0) | 31(10.0) | 0.482 |
| Alpha-glucosidase inhibitor | 68(35.6) | 83(26.9) | **0.039** |
| **Angiographic Characteristics** | | | |
| Dominance artery (right) | 183(95.8) | 305(98.7) | 0.079 |
| **Number of Diseased Vessels** | | | |
| 1 | 5(2.6) | 89(28.8) | **0.000** |
| 2 | 61(31.9) | 140(45.3) | **0.003** |
| 3 | 125(65.4) | 80(25.9) | **0.000** |
| Syntax score | 23.00(20.00-25.00) | 19.00(12.00-21.00) | **0.000** |
| Rentrop grade≥2 | 119(81.5) | 249(91.5) | **0.003** |
| Abrupt stump | 69(47.3) | 136(50.0) | 0.593 |
| Calcification | 35(24.0) | 45(16.5) | 0.066 |
| Bending ≥45° | 129(88.4) | 232(85.3) | 0.384 |
| CTO length≥20mm | 117(80.1) | 191(70.2) | **0.028** |
| Values are n(%), mean±SD or median with interquartile range.  PCI: percutaneous transluminal coronary intervention; MT: medical therapy; CABG: coronary artery bypass grafting; PVD: peripheral vascular disease; MI: myocardial infarction; CKD: Chronic kidney disease; COPD: Chronic obstructive pulmonary disease; LVEF: left ventricular ejection fraction; BMI: body mass index; CCB: calcium-channel blocker; ACEI/ARB: angiotensin converting enzyme inhibitor/angiotensin-receptor blocker; CTO: chronic total occlusion; HF: heart failure; RWMA: reginal wall motion abnormality. | | | |

| **Table S9 Clinical Outcomes (CTO-CABG versus successful CTO-PCI)** | | | |
| --- | --- | --- | --- |
|  | CTO-CABG | Successful CTO-PCI | P value |
| **All cause death** |  |  |  |
| Event per 1000 patient-years | 14.60 | 10.44 | 0.354 |
| Unadjusted HR (95% CI) | 1 | 0.727(0.354-1.494) | 0.386 |
| Adjusted HR (95% CI) | 1 | 0.540(0.234-1.243) | 0.147 |
| **Noncardiac death** |  |  |  |
| Event per 1000 patient-years | 6.81 | 4.18 | 0.373 |
| Unadjusted HR (95% CI) | 1 | 0.616(0.207-1.838) | 0.385 |
| Adjusted HR (95% CI) | 1 | 0.375(0.094-1.506) | 0.167 |
| **Cardiac death** |  |  |  |
| Event per 1000 patient-years | 7.78 | 6.26 | 0.653 |
| Unadjusted HR (95% CI) | 1 | 0.827(0.316-2.165) | 0.699 |
| Adjusted HR (95% CI) | 1 | 0.576(0.193-1.717) | 0.322 |
| **Probable/Definite cardiac death** |  |  |  |
| Event per 1000 patient-years | 3.89 | 3.48 | 0.868 |
| Unadjusted HR (95% CI) | 1 | 0.982(0.260-3.715) | 0.979 |
| Adjusted HR (95% CI) | 1 | 0.541(0.119-2.449) | 0.425 |
| **Repeat nonfatal MI** |  |  |  |
| Event per 1000 patient-years | 3.93 | 9.21 | 0.124 |
| Unadjusted HR (95% CI) | 1 | 2.616(0.843-8.112) | 0.096 |
| Adjusted HR (95% CI) | 1 | 2.991(0.813-11.005) | 0.099 |
| **Repeat revascularization** |  |  |  |
| Event per 1000 patient-years | 11.14 | 59.74 | **0.000** |
| Unadjusted HR (95% CI) | 1 | **5.051(2.665-9.575)** | **0.000** |
| Adjusted HR (95% CI) | 1 | **4.459(2.044-9.726)** | **0.000** |
| **TVR** |  |  |  |
| Event per 1000 patient-years | 1.97 | 35.20 | **0.000** |
| Unadjusted HR (95% CI) | 1 | **16.160(3.913-66.746)** | **0.000** |
| Adjusted HR (95% CI) | 1 | **21.676(2.896-162.24)** | **0.003** |
| Adjusted covariates (model 2): age, sex, PVD, CKD, COPD, prior MI, systolic HF, LVEF, reginal wall motion abnormality, single vessel disease, triple-vessel disease, syntax scores and HbA1c.  HR: hazard ratio; CI: conference interval; other abbreviations as in **Table 1**. | | | |

| **Table S10 Baseline Characteristics (Initial CTO-MT versus CTO-CABG)** | | | |
| --- | --- | --- | --- |
|  | Initial CTO-MT (n=233) | CTO-CABG (n=191) | P value |
| **Clinical Characteristics** | | | |
| Age (yrs) | 62.19±10.08 | 61.43±8.17 | 0.396 |
| Male | 160(68.7) | 147(77.0) | 0.057 |
| Hypertension | 155(66.5) | 131(68.6) | 0.652 |
| Dyslipidemia | 78(33.5) | 37(19.4) | **0.001** |
| PVD | 9(3.9) | 4(2.1) | 0.293 |
| Prior MI | 127(54.5) | 121(63.4) | 0.066 |
| Prior PCI | 49(21.0) | 16(8.4) | **0.000** |
| Prior stroke | 19(8.2) | 15(7.9) | 0.910 |
| **Heart failure** | 74(31.8) | 80(41.9) | **0.031** |
| Systolic heart failure | 38(16.3) | 40(20.9) | 0.221 |
| Diastolic heart failure | 36(15.5) | 40(20.9) | 0.142 |
| CKD | 13(5.6) | 7(3.7) | 0.355 |
| COPD /asthma | 2(0.9) | 2(1.0) | 1.000 |
| Hyperuricemia | 56(24.0) | 38(19.9) | 0.307 |
| Smoking | 112(48.1) | 95(49.7) | 0.732 |
| Drinking | 38(16.3) | 36(18.8) | 0.493 |
| BMI (kg/m^2^) | 26.23±3.39 | 26.57±2.89 | 0.264 |
| LVEF (%) | 61.00(53.00-66.00) | 59.00(52.00-64.00) | **0.013** |
| RWMA | 79(33.9) | 86(45.0) | **0.019** |
| Fasting blood glucose(mmol/L) | 7.12(6.23-9.12) | 7.85(6.13-9.37) | 0.583 |
| HbA1c(%) | 7.30(6.80-8.00) | 7.30(7.10-7.43) | 0.245 |
| **Medical Treatment** | | | |
| Aspirin | 227(97.4) | 182(96.3) | 0.505 |
| P2Y_12_ inhibitor | 206(88.4) | 106(56.1) | **0.000** |
| Statin | 226(97.0) | 161(85.2) | **0.000** |
| Nitrites | 120(51.5) | 78(41.3) | **0.036** |
| Beta-blocker | 174(74.7) | 161(85.2) | **0.008** |
| CCB | 67(28.8) | 50(26.5) | 0.600 |
| ACEI/ARB | 136(58.4) | 102(54.0) | 0.365 |
| Insulin | 82(35.2) | 68(35.6) | 0.930 |
| Sulfonylureas | 33(14.2) | 31(16.2) | 0.554 |
| Glinide | 8(3.4) | 7(3.7) | 0.898 |
| Biguanides | 79(33.9) | 72(37.7) | 0.417 |
| Thiazolidinediones | 21(9.0) | 23(12.0) | 0.309 |
| Alpha-glucosidase inhibitor | 87(37.3) | 68(35.6) | 0.712 |
| **Angiographic Characteristics** | | | |
| Dominance artery (right) | 214(91.8) | 183(95.8) | 0.096 |
| **Number of Diseased Vessels** | | | |
| 1 | 48(20.6) | 5(2.6) | **0.000** |
| 2 | 80(34.3) | 61(31.9) | 0.602 |
| 3 | 105(45.1) | 125(65.4) | **0.000** |
| Syntax score | 20.00(17.00-27.00) | 23.00(20.00-25.00) | 0.102 |
| Rentrop grade≥2 | 168(85.7) | 119(81.5) | 0.295 |
| Abrupt stump | 111(56.6) | 69(47.3) | 0.086 |
| Calcification | 40(20.4) | 35(24.0) | 0.431 |
| Bending ≥45° | 170(86.7) | 129(88.4) | 0.655 |
| CTO length≥20mm | 146(74.5) | 117(80.1) | 0.220 |
| Values are n(%), mean±SD or median with interquartile range.  PCI: percutaneous transluminal coronary intervention; MT: medical therapy; CABG: coronary artery bypass grafting; PVD: peripheral vascular disease; MI: myocardial infarction; CKD: Chronic kidney disease; COPD: Chronic obstructive pulmonary disease; LVEF: left ventricular ejection fraction; BMI: body mass index; CCB: calcium-channel blocker; ACEI/ARB: angiotensin converting enzyme inhibitor/angiotensin-receptor blocker; CTO: chronic total occlusion; HF: heart failure; RWMA: reginal wall motion abnormality. | | | |

| **Table S11 Clinical Outcomes (Initial CTO-MT versus CTO-CABG)** | | | |
| --- | --- | --- | --- |
|  | Initial CTO-MT | CTO-CABG | P value |
| **All cause death** |  |  |  |
| Event per 1000 patient-years | 32.01 | 14.60 | **0.009** |
| Unadjusted HR (95% CI) | 1 | **0.456(0.246-0.848)** | **0.013** |
| Adjusted HR (95% CI) | 1 | 0.511(0.258-1.013) | 0.054 |
| **Noncardiac death** |  |  |  |
| Event per 1000 patient-years | 7.23 | 6.81 | 0.910 |
| Unadjusted HR (95% CI) | 1 | 0.821(0.287-2.353) | 0.714 |
| Adjusted HR (95% CI) | 1 | 1.959(0.429-8.958) | 0.386 |
| **Cardiac death** |  |  |  |
| Event per 1000 patient-years | 24.79 | 7.78 | **0.002** |
| Unadjusted HR (95% CI) | 1 | **0.335(0.150-0.747)** | **0.008** |
| Adjusted HR (95% CI) | 1 | **0.355(0.146-0.863)** | **0.022** |
| **Probable/Definite cardiac death** |  |  |  |
| Event per 1000 patient-years | 13.43 | 3.89 | **0.020** |
| Unadjusted HR (95% CI) | 1 | **0.322(0.105-0.989)** | **0.048** |
| Adjusted HR (95% CI) | 1 | 0.457(0.136-1.537) | 0.206 |
| **Repeat nonfatal MI** |  |  |  |
| Event per 1000 patient-years | 16.95 | 3.93 | **0.004** |
| Unadjusted HR (95% CI) | 1 | **0.200(0.066-0.605)** | **0.004** |
| Adjusted HR (95% CI) | 1 | **0.150(0.043-0.530)** | **0.003** |
| **Repeat revascularization** |  |  |  |
| Event per 1000 patient-years | 47.89 | 11.14 | **0.000** |
| Unadjusted HR (95% CI) | 1 | **0.244(0.125-0.473)** | **0.000** |
| Adjusted HR (95% CI) | 1 | **0.207(0.093-0.464)** | **0.000** |
| **TVR** |  |  |  |
| Event per 1000 patient-years | 30.49 | 1.97 | **0.000** |
| Unadjusted HR (95% CI) | 1 | **0.071(0.017-0.296)** | **0.000** |
| Adjusted HR (95% CI) | 1 | **0.046(0.006-0.348)** | **0.003** |
| Adjusted covariates (model 2): age, sex, PVD, CKD, COPD, prior MI, systolic HF, LVEF, reginal wall motion abnormality, single vessel disease, triple-vessel disease, syntax scores and HbA1c.  HR: hazard ratio; CI: conference interval; other abbreviations as in **Table 1**. | | | |

| **Table S12 Baseline Characteristics (Failed CTO-PCI versus CTO-CABG)** | | | |
| --- | --- | --- | --- |
|  | Failed CTO-PCI (n=) | CTO-CABG (n=191) | P value |
| **Clinical Characteristics** | | | |
| Age (yrs) | 58.73±11.09 | 61.43±8.17 | **0.007** |
| Male | 151(79.1) | 147(77.0) | 0.621 |
| Hypertension | 138(72.3) | 131(68.6) | 0.433 |
| Dyslipidemia | 72(37.7) | 37(19.4) | **0.000** |
| PVD | 7(3.7) | 4(2.1) | 0.359 |
| Prior MI | 97(50.8) | 121(63.4) | **0.013** |
| Prior PCI | 41(21.5) | 16(8.4) | **0.000** |
| Prior stroke | 14(7.3) | 15(7.9) | 0.847 |
| **Heart failure** | 47(24.6) | 80(41.9) | **0.000** |
| Systolic heart failure | 11(5.8) | 40(20.9) | **0.000** |
| Diastolic heart failure | 36(18.8) | 40(20.9) | 0.608 |
| CKD | 2(1.0) | 7(3.7) | 0.174 |
| COPD /asthma | 2(1.0) | 2(1.0) | 1.000 |
| Hyperuricemia | 39(20.4) | 38(19.9) | 0.899 |
| Smoking | 96(50.3) | 95(49.7) | 0.918 |
| Drinking | 33(17.3) | 36(18.8) | 0.690 |
| BMI (kg/m^2^) | 26.69±3.21 | 26.57±2.89 | 0.710 |
| LVEF (%) | 62.00(56.00-66.00) | 59.00(52.00-64.00) | **0.000** |
| RWMA | 57(29.8) | 86(45.0) | **0.002** |
| Fasting blood glucose(mmol/L) | 7.30(6.32-9.39) | 7.85(6.13-9.37) | 0.926 |
| HbA1c(%) | 7.30(6.60-8.30) | 7.30(7.10-7.43) | 0.683 |
| **Medical Treatment** | | | |
| Aspirin | 184(96,3) | 182(96.3) | 0.984 |
| P2Y_12_ inhibitor | 179(93.7) | 106(56.1) | **0.000** |
| Statin | 181(94.8) | 161(85.2) | **0.002** |
| Nitrites | 136(71.2) | 78(41.3) | **0.000** |
| Beta-blocker | 153(80.1) | 161(85.2) | 0.191 |
| CCB | 53(27.7) | 50(26.5) | 0.777 |
| ACEI/ARB | 104(54.5) | 102(54.0) | 0.925 |
| Insulin | 71(37.2) | 68(35.6) | 0.750 |
| Sulfonylureas | 28(14.7) | 31(16.2) | 0.671 |
| Glinide | 11(5.8) | 7(3.7) | 0.334 |
| Biguanides | 65(34.0) | 72(37.7) | 0.455 |
| Thiazolidinediones | 26(13.6) | 23(12.0) | 0.646 |
| Alpha-glucosidase inhibitor | 62(32.5) | 68(35.6) | 0.517 |
| **Angiographic Characteristics** | | | |
| Dominance artery (right) | 182(95.3) | 183(95.8) | 0.804 |
| **Number of Diseased Vessels** | | | |
| 1 | 67(35.1) | 5(2.6) | **0.000** |
| 2 | 67(35.1) | 61(31.9) | 0.515 |
| 3 | 57(29.8) | 125(65.4) | **0.000** |
| Syntax score | 19.00(12.00-27.00) | 23.00(20.00-25.00) | **0.000** |
| Rentrop grade≥2 | 129(78.7) | 119(81.5) | 0.531 |
| Abrupt stump | 69(42.1) | 69(47.3) | 0.359 |
| Calcification | 31(18.9) | 35(24.0) | 0.276 |
| Bending ≥45° | 144(87.8) | 129(88.4) | 0.881 |
| CTO length≥20mm | 124(75.6) | 117(80.1) | 0.339 |
| Values are n(%), mean±SD or median with interquartile range.  PCI: percutaneous transluminal coronary intervention; MT: medical therapy; CABG: coronary artery bypass grafting; PVD: peripheral vascular disease; MI: myocardial infarction; CKD: Chronic kidney disease; COPD: Chronic obstructive pulmonary disease; LVEF: left ventricular ejection fraction; BMI: body mass index; CCB: calcium-channel blocker; ACEI/ARB: angiotensin converting enzyme inhibitor/angiotensin-receptor blocker; CTO: chronic total occlusion; HF: heart failure; RWMA: reginal wall motion abnormality. | | | |

| **Table S13 Clinical Outcomes (Failed CTO-PCI versus CTO-CABG)** | | | |
| --- | --- | --- | --- |
|  | Failed CTO-PCI | CTO-CABG | **P value** |
| **All cause death** |  |  |  |
| Event per 1000 patient-years | 25.39 | 14.60 | 0.094 |
| Unadjusted HR (95% CI) | 1 | 0.537(0.275-1.048) | 0.068 |
| Adjusted HR (95% CI) | 1 | **0.322(0.130-0.800)** | **0.015** |
| **Noncardiac death** |  |  |  |
| Event per 1000 patient-years | 4.84 | 6.81 | 0.582 |
| Unadjusted HR (95% CI) | 1 | 1.188(0.343-4.113) | 0.786 |
| Adjusted HR (95% CI) | 1 | 2.120(0.357-12.592) | 0.408 |
| **Cardiac death** |  |  |  |
| Event per 1000 patient-years | 20.56 | 7.78 | **0.018** |
| Unadjusted HR (95% CI) | 1 | **0.372(0.160-0.866)** | **0.022** |
| Adjusted HR (95% CI) | 1 | **0.146(0.044-0.483)** | **0.002** |
| **Probable/Definite cardiac death** |  |  |  |
| Event per 1000 patient-years | 10.88 | 3.89 | 0.073 |
| Unadjusted HR (95% CI) | 1 | 0.363(0.111-1.182) | 0.092 |
| Adjusted HR (95% CI) | 1 | 0.223(0.047-1.064) | 0.060 |
| **Repeat nonfatal MI** |  |  |  |
| Event per 1000 patient-years | 11.21 | 3.93 | 0.067 |
| Unadjusted HR (95% CI) | 1 | 0.352(0.108-1.152) | 0.084 |
| Adjusted HR (95% CI) | 1 | 0.472(0.113-1.975) | 0.304 |
| **Repeat revascularization** |  |  |  |
| Event per 1000 patient-years | 76.80 | 11.14 | **0.000** |
| Unadjusted HR (95% CI) | 1 | **0.160(0.083-0.308)** | **0.000** |
| Adjusted HR (95% CI) | 1 | **0.174(0.074-0.406)** | **0.000** |
| **TVR** |  |  |  |
| Event per 1000 patient-years | 48.21 | 1.97 | **0.000** |
| Unadjusted HR (95% CI) | 1 | **0.048(0.011-0.198)** | **0.000** |
| Adjusted HR (95% CI) | 1 | **0.029(0.004-0.230)** | **0.001** |
| Adjusted covariates (model 2): age, sex, PVD, CKD, COPD, prior MI, systolic HF, LVEF, reginal wall motion abnormality, single vessel disease, triple-vessel disease, syntax scores and HbA1c.  HR: hazard ratio; CI: conference interval; other abbreviations as in **Table 1**. | | | |

| 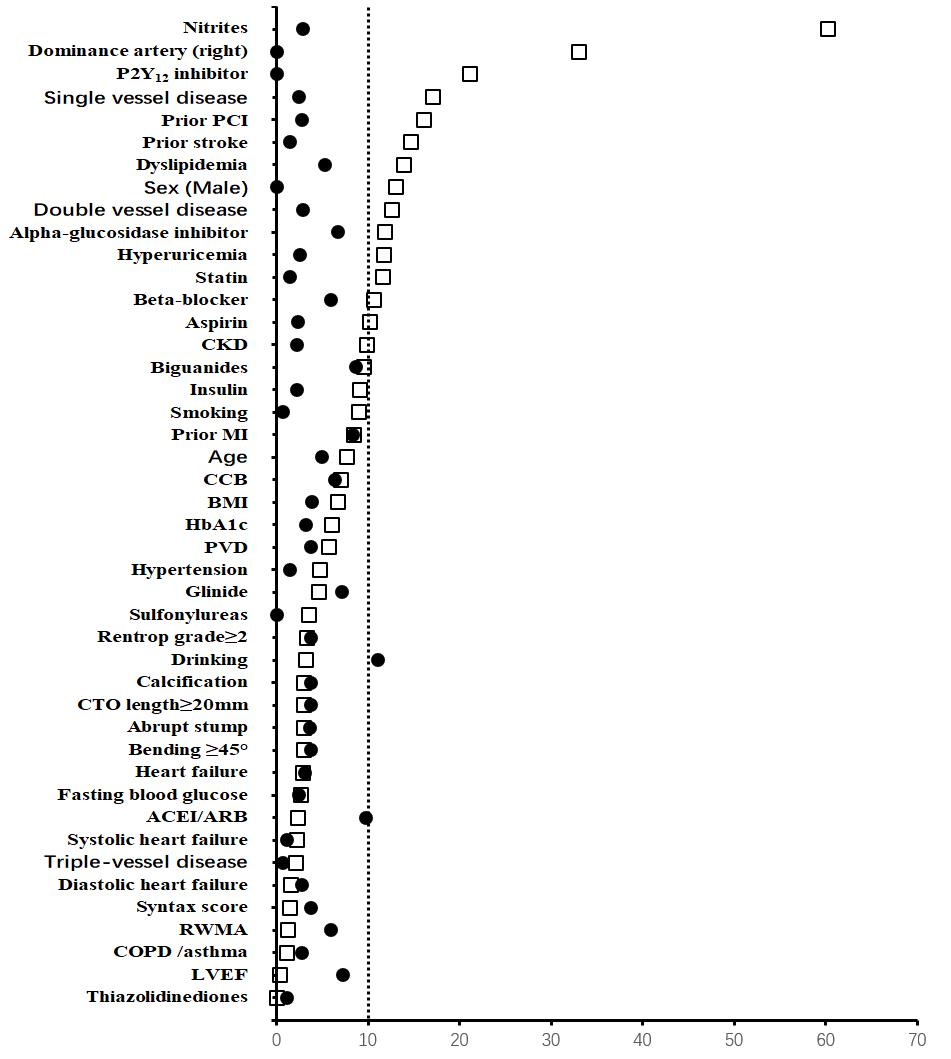 |
| --- |
| **Figure S1 Absolute Standard Difference before and after Propensity Matching** |
